# Explainable ViT-XGB: A hybrid transformer-boosting approach for multiclass diabetic retinopathy grading

**DOI:** 10.1016/j.jgeb.2026.100848

**Published:** 2026-11-04

**Authors:** Azhar Imran, Sun-Hee Kim, Jahanzaib Latif, Raja Majid Mehmood

**Affiliations:** a Centre for Advanced Analytics, COE for Artificial Intelligence, Multimedia University, Persiaran Multimedia, Cyberjaya 63100, Selangor, Malaysia; b College of Computer Computer Science, Beijing University of Technology, 100124, Beijing, China; c Department of Software, SeoKyeong University, Seoul, 02713, South Korea; d Department of Computer Science & IT, University of Lahore, Sargodha Campus, Pakistan; e Faculty of Computing and Informatics, Multimedia University, Persiaran Multimedia, Cyberjaya 63100, Selangor, Malaysia

**Keywords:** Diabetic retinopathy, Fundus imaging, Vision Transformer, Gradient boosting, SMOTE, Grad-CAM, Explainable AI

## Abstract

Diabetic retinopathy (DR) is a leading cause of vision impairment worldwide, necessitating accurate and scalable automated screening systems. Despite recent advances in deep learning, reliable multiclass DR grading remains challenging due to subtle lesion characteristics, severe class imbalance, and limited model interpretability. In this study, we propose ViT-XGB, a hybrid framework that combines the global representation capability of a pretrained Vision Transformer (ViT) with the robustness of Extreme Gradient Boosting (XGBoost) for multiclass DR classification. The ViT model extracts high-level contextual features from fundus images, which are subsequently used to train an XGBoost classifier. To address class imbalance, the Synthetic Minority Oversampling Technique (SMOTE) is applied in the latent feature space of the training partition only, so that every validation and test image remains a real, unaltered fundus photograph. Furthermore, Grad-CAM-based explainability is incorporated to provide visual evidence of the retinal regions that drive the shared ViT representation. The proposed framework is evaluated on 6904 fundus images (3662 from APTOS 2019 and 3242 from the Kaggle 2015 EyePACS set) using a stratified 80/10/10 split. On the held-out test set of 691 images, ViT-XGB reached an accuracy of 98.52% (95% confidence interval, 97.31% to 99.19%), a Cohen’s kappa of 0.978, and a macro F1-score of 97.72%. These figures come from an internal test split drawn from the same two sources. The pipeline also carries a notable computational cost: ViT fine-tuning requires a GPU, and the t-SNE stage is non-parametric, so it must be refitted or approximated before it can process new images one at a time. Independent external validation is therefore needed before the approach can support large-scale DR screening and teleophthalmology applications.

## 1 Introduction

Diabetic retinopathy (DR) is the leading microvascular complication of diabetes mellitus and has become the main cause of visual loss and blindness in the working age population worldwide.^1^ The pathological development of DR is associated with long-term hyperglycemia-induced injury to the retinal vasculature, leading to inflammation, vascular permeability, capillary occlusion, and retinal ischemia.^2^ Clinically, DR is a disorder with a well-defined range of severity stages, beginning with non-proliferative DR (NPDR), which is divided into Mild, Moderate, and Severe stages based on the presence of retinal lesions such as microaneurysms, retinal hemorrhages, venous beading, and intraretinal microvascular abnormalities. It progresses to proliferative DR (PDR), marked by the development of abnormal new blood vessels (neovascularization), which pose a substantial risk for vitreous hemorrhage, fibrosis, and tractional retinal detachment.^3^

The worldwide epidemiological burden of DR is an important public health concern. According to the International Diabetes Federation, there were 537 million adults with diabetes in 2021; by 2030, this number is predicted to rise to 643 million, and by 2045, it will reach 783 million.^4^^,^^5^ Epidemiological studies have estimated that DR is present in one-third of patients with diabetes, and 10% of them progress to vision-threatening stages.^6^ Thus, there is a need for organized screening for diabetic retinopathy because of its progressive nature and the lack of symptoms until advanced or irreparable loss of vision occurs. In clinical workflows, diabetic retinopathy screening depends on the manual assessment of color fundus photographs by expert ophthalmologists or retinal specialists.^7^ Nevertheless, the conventional method suffers from several limitations: it requires a tremendous amount of labor and is time-consuming, the findings are affected by inter- and intra-observer variability, and it is not sustainable with the increasing prevalence of diabetes worldwide. Such constraints have spurred considerable research interest in the design of automated, high-accuracy, and scalable solutions based on artificial intelligence for the screening and severity classification of diabetic retinopathy.

Deep learning, and convolutional neural networks (CNNs) in particular, has automated much of DR detection, yet three problems still limit its clinical use.^8^^,^^9^ First, severity grading is a fine-grained task. Neighboring grades may differ by only a few small lesions, so a model needs features that capture both tiny local changes and how those changes are distributed across the retina. Second, screening datasets are heavily imbalanced. No DR and Mild DR images far outnumber Severe DR and PDR images, which biases models toward the common grades and away from the cases that most need referral. Third, clinicians are reluctant to act on predictions they cannot inspect, so a grading model should expose the evidence behind its output.

To address these problems, we present ViT-XGB, a hybrid model that uses a Vision Transformer (ViT) as a feature extractor and XGBoost as the classifier. Self-attention lets the ViT relate lesions that lie far apart in the fundus image, while gradient-boosted trees are well suited to the compact, tabular feature vectors that the ViT produces. Among boosting libraries, we chose XGBoost^10^ over LightGBM^11^ and CatBoost^12^ for practical reasons. Our inputs are dense, continuous features with no categorical variables, so the main strength of CatBoost, its ordered handling of categorical data, does not apply. The training table is small by the standards of tree ensembles (about 15,600 vectors after balancing), so the speed advantage of LightGBM on very large tables matters little. XGBoost, in turn, offers explicit L1 and L2 penalties on leaf weights, a minimum split gain, and second-order split scoring, which we used to control model complexity, and it is the booster most often used in earlier hybrid DR studies,[13], [14], [15] which eases comparison. Benchmarks on tabular data show that the three libraries usually perform within a narrow margin of one another,^16^^,^^17^ so our choice reflects fit to the data rather than a claim that XGBoost is better in general. Pairing a deep feature extractor with boosted trees also has precedent in medical imaging. CNN features combined with XGBoost have been used for DR detection,^13^ and ViT features have recently been combined with XGBoost for colorectal carcinoma recognition in colonoscopy images.^18^ This combination has received little attention for five-class DR grading, particularly together with feature-space balancing and visual explanation.

The main contributions of this study are as follows.

• A novel hybrid framework, ViT-XGB, is proposed for five-class diabetic retinopathy (DR) grading, combining transformer-based feature extraction with gradient boosting for improved classification performance.
• A feature-space class balancing strategy based on SMOTE, applied to the training features only, is introduced to address severe class imbalance and enhance recognition of minority DR categories.
• Grad-CAM heatmaps computed through the shared ViT backbone are incorporated to provide visual evidence of the retinal regions behind each prediction, improving transparency and trust in model predictions.
• Experiments on 6904 images from two public datasets, with an ablation study, 95% confidence intervals, and paired significance testing, show that the proposed pipeline compares favorably with CNN, ViT, and ViT-hybrid baselines trained under the same protocol.

## 2 Literature review

As diabetes continues to rise globally, diabetic retinopathy has emerged as the second most common cause of avoidable blindness, highlighting the demand for efficient and large-scale screening methods.^19^ Ophthalmologists have traditionally manually examined color fundus photographs for this purpose in current clinical practice; however, this process is time-consuming, resource-intensive, and prone to variability between clinical observers.^20^ This has spurred a lot of research on automated and AI-based solutions for DR detection and grading. The evolution of such solutions ranges from classical ML to DL methods and, more recently, to transformer-driven models.[21], [22], [23] Traditional ML-based early automated DR systems require a multistage pipeline, including lesion segmentation and hand-crafted feature engineering. Dedicated algorithms are also available for detecting particular pathologies, such as microaneurysms, using morphological operations and circular Hough transforms; hemorrhages, using region-growing methods; and exudates, using intensity-based thresholding.^24^ The handcrafted features were then classified using classifiers, including DT, RF, and SVM, for both binary detection (DR vs. normal) and multi-class grading according to the International Clinical Diabetic Retinopathy scale.[25], [26], [27] Although crude, these methods were constrained by the accuracy of the handcrafted features and were not capable of modeling the diversity of the appearance and complexity of retinal lesions.

Recent advances in deep learning, particularly convolutional neural networks (CNNs), have significantly advanced automated diabetic retinopathy (DR) detection by enabling hierarchical feature learning directly from raw fundus images, eliminating the need for handcrafted features.^28^ Landmark work by Gulshan et al. ^29^ demonstrated expert-level performance of CNNs in identifying referable DR, establishing their suitability for large-scale screening. Subsequent studies, including those inspired by the Kaggle 2015 DR challenge, further improved performance through enhanced preprocessing, data augmentation, and ensemble strategies.^30^ Transfer learning using pretrained architectures such as VGGNet, ResNet, InceptionV3, DenseNet, and EfficientNet has been widely adopted to improve classification accuracy.[31], [32], [33], [34], [35], [36] Hybrid approaches combining CNN-based feature extraction with traditional machine learning classifiers, particularly XGBoost, have also shown strong performance, highlighting the complementary strengths of deep feature representation and boosting-based classification.[13], [14], [15] Ensemble strategies that combine several learners have also been explored, using both image-derived features and clinical or biochemical variables.[37], [38], [39], [40] DR classification has been extended to fine-grained multiclass settings. Ensemble and hybrid models integrating CNNs with sequence models or boosting techniques have shown promising results, achieving accuracies above 95% on benchmark datasets such as APTOS 2019.^41^ Other studies have explored patch-based learning, lesion localization, and pretrained CNN variants, typically reporting performance in the range of 84% to 87% for five-class classification.[42], [43], [44] Transformer-based models have also emerged as a competitive alternative, leveraging global attention mechanisms to improve representation learning, with recent approaches achieving accuracies up to 95%.^45^^,^^46^ Despite these advances, achieving robust, interpretable, and imbalance-aware multiclass DR grading remains an open challenge.

**Fig. 1:**
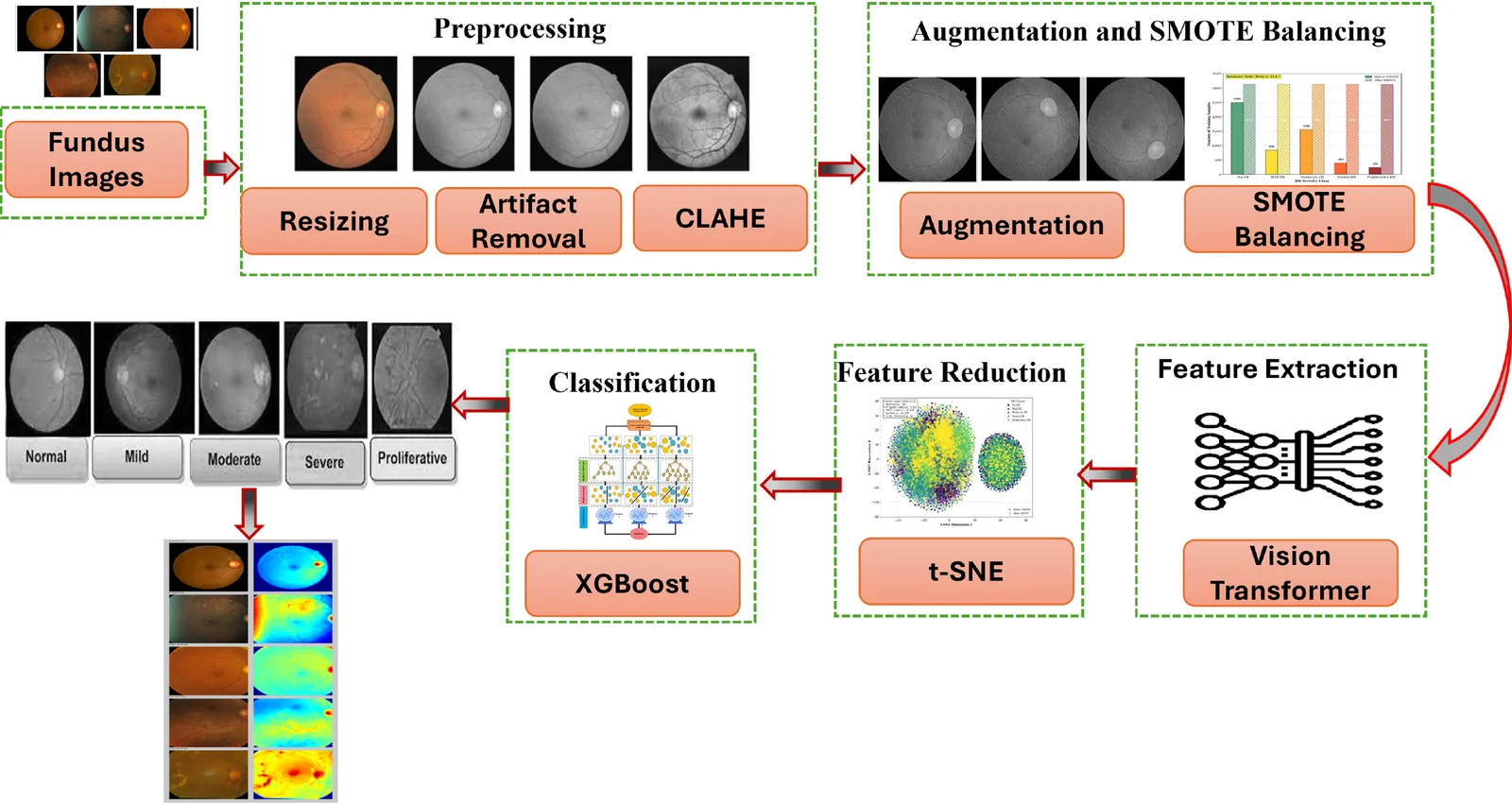
Overview of the proposed ViT-XGB framework for diabetic retinopathy severity grading, illustrating image input, preprocessing, data augmentation, SMOTE balancing (applied to the extracted ViT feature vectors of the training partition), ViT-based feature extraction, feature reduction using t-SNE, XGBoost classification into five severity levels (normal, mild, moderate, severe, PDR), and Grad-CAM-based visual interpretation.

CNNs are generally limited by their local receptive fields, but this needs qualification. In principle, the receptive field of a deep CNN covers the whole image after enough layers. In practice, the effective receptive field is concentrated near its center and grows slowly with depth.^47^ Recent designs have narrowed this gap. Channel and spatial attention modules such as squeeze-and-excitation and CBAM,^48^^,^^49^ large-kernel networks such as ConvNeXt,^50^ and attention mechanisms designed for DR^51^^,^^52^ all widen the context that a CNN can use. The difference between the two families is therefore one of inductive bias rather than a hard limit. CNNs favor local texture and build global context step by step, whereas ViTs relate all image patches from the first layer onward and keep more global information in their intermediate representations.^53^ This distinction matters for DR because severity depends on how lesions are spread across the retina, not only on whether a lesion is present. The 4-2-1 rule for severe NPDR, for example, depends on hemorrhages in all four quadrants, venous beading in two or more quadrants, or intraretinal microvascular abnormalities in at least one quadrant.^54^

Transformers have accordingly been applied to retinal disease with growing success. Examples include ViT-DR,^23^ the ViT-based grading model of Wu et al.,^55^ a ViT with residual attention,^56^ lesion-aware transformers,^57^ the multiple-instance MIL-ViT,^58^ image transformers evaluated across several fundus datasets,^59^ and OcuViT for DR and age-related macular degeneration.^45^ RETFound, a foundation model pretrained on 1.6 million unlabeled retinal images, further showed that transformer encoders adapt well to a range of retinal diseases.^60^ Two issues remain. ViTs need more training data than CNNs to reach their potential,^61^^,^^62^ which is a concern for DR datasets of a few thousand images. In addition, most of the studies above train the transformer end to end and leave class imbalance to the loss function or to image-level augmentation. Taken together, these studies point to three gaps. First, CNN-based graders, even with attention, must learn long-range relations indirectly, while ViTs model them directly but are data hungry. Second, class imbalance is usually handled at the image level, through weighted losses or GAN-based augmentation,^63^^,^^64^ and balancing in a learned feature space has received little attention. Third, explanations, where provided, are often limited to a few illustrative heatmaps.^65^^,^^66^ To bridge these gaps, this study keeps a pretrained ViT as a global feature extractor, balances the classes in its feature space, and assigns the final decision to XGBoost, which is well suited to compact feature vectors and small training sets. Grad-CAM is then used to check whether the evidence the backbone relies on matches known lesions.

## 3 Materials and Methods

The overall process of the proposed Vision Transformer-XGBoost (ViT-XGB) model for multiclass classification of diabetic retinopathy is shown in Fig. 1 This study began with the acquisition and preprocessing of fundus images from two publicly accessible datasets: APTOS 2019 Blindness Detection^67^ and Kaggle 2015 Diabetic Retinopathy Detection.^30^^,^^68^ A comprehensive preprocessing pipeline was employed to enhance the quality of the images and focus on patterns with clinical significance. In particular, contrast-limited adaptive histogram equalization (CLAHE) was applied to increase the local contrast and detectability of faint retinal lesions.^69^ To satisfy the input size requirements of the pretrained ViT, all images were scaled to 224 × 224. Various forms of augmentation were performed to augment the diversity of the data and thus enhance the generalization ability of the model, such as horizontal and vertical flipping, small rotations (±15°), and brightness and contrast adjustments. The dataset was stratified and then split into training (80%), validation (10%), and test sets (10%). The split was made before any model development, and the test partition was used only once, for the final evaluation. After the data were preprocessed, a pretrained ViT-B/16 model was adopted as a feature extractor. The model treats every image as a sequence of 16 × 16 non-overlapping image patches that are then linearly embedded and passed through a cascade of transformer encoder layers, producing high-dimensional features that represent the local and global contextual information of retinal pathology.

To address the extreme label imbalance in the data, the ViT-based feature vectors of the training partition were passed through the Synthetic Minority Oversampling Technique (SMOTE)^70^ to generate synthetic samples for minority classes and obtain a balanced training set. Subsequently, the high-dimensional feature representations were mapped into a low-dimensional space using t-distributed stochastic neighbor embedding (t-SNE), which is a nonlinear dimensionality reduction technique that preserves the local neighborhood structure of the data and reduces computational complexity for later classification.^71^^,^^72^ The balanced and dimension-reduced feature vectors were then employed to train an XGBoost classifier for five-class DR severity classification, where XGBoost is an iterative gradient-boosting decision tree-based algorithm that builds an ensemble of decision trees. By incorporating regularization terms into the objective function, XGBoost regulates the complexity, overfitting, and generalization of the model. To improve interpretability and facilitate clinical utilization, model predictions were subsequently interpreted using Gradient-weighted Class Activation Mapping (Grad-CAM), which generates heat maps that visually identify areas within the retina that contribute most to the classification results, allowing verification of clinically relevant lesion localization.

### 3.1 Dataset acquisition

This study used two publicly available fundus datasets. The first is the labeled training set of the APTOS 2019 Blindness Detection challenge,^67^ which contains 3662 color fundus photographs collected by Aravind Eye Hospital in India. The images were graded by clinicians on the International Clinical Diabetic Retinopathy (ICDR) scale,^54^ from 0 (No DR) to 4 (PDR). The second source is the Kaggle 2015 Diabetic Retinopathy Detection dataset,^68^ provided by the EyePACS telescreening program.^73^ Its labeled training partition contains 35,126 images graded on the same scale. We added 3242 images from the Kaggle training partition to the 3662 APTOS images, giving 6904 images in total. We used a subset rather than the full Kaggle set for three reasons. First, the full set is about ten times larger than APTOS 2019, and 73.5% of its images are graded No DR, so adding all of it would have made the merged data even more skewed toward No DR. Second, many Kaggle 2015 images have poor illumination or camera artifacts; we retained only images that passed a basic quality screen. Third, fine-tuning ViT-B/16 on more than 38,000 images was beyond the memory and time budget of the laptop GPU used in this work. Both datasets are de-identified and publicly released for research. Neither provides patient demographics such as age, sex, or ethnicity. The merged dataset was split into training (80%), validation (10%), and test (10%) partitions using stratified sampling, so that each partition keeps the same class proportions. Table 1 lists the number of images per grade in every partition. The class distribution remains skewed toward No DR, as expected in screening populations, and representative images of each grade are shown in Fig. 2.

**Fig. 2:**
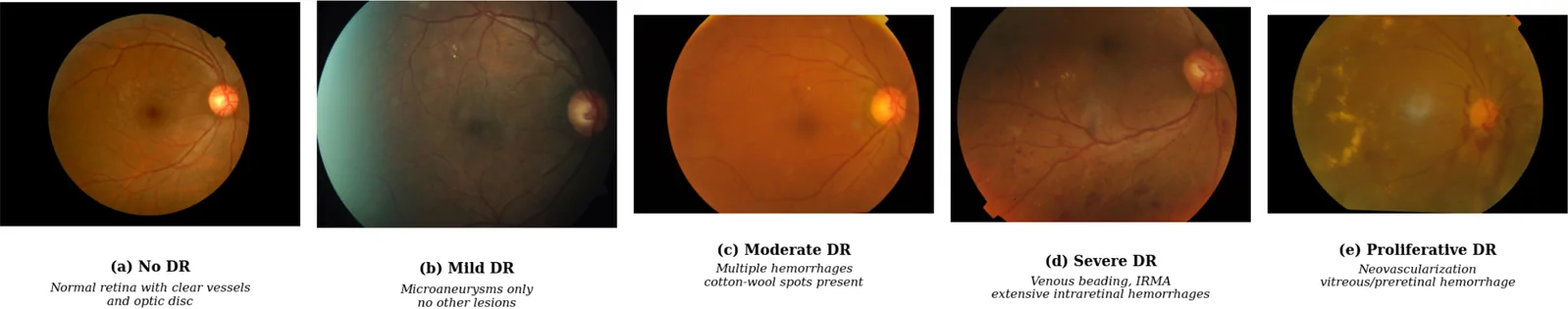
Examples of the five diabetic retinopathy (DR) severity levels: (a) No DR: normal retina with clear vessels and an optic disc; (b) Mild DR: presence of microaneurysms only; (c) Moderate DR: multiple hemorrhages and cotton-wool spots; (d) Severe DR: venous beading, intraretinal microvascular abnormalities (IRMA), and extensive hemorrhages; (e) Proliferative DR: neovascularization with vitreous or preretinal hemorrhage.

**Table 1:** Number of images per DR grade in each partition of the merged dataset (APTOS 2019: 3662 images; Kaggle 2015 subset: 3242 images). SMOTE was applied to the training feature vectors only; the validation and test partitions contain real images only.

DR severity class	Training	Training	Oversampling	Validation	Test	Total
	(original)	after SMOTE	ratio			
0: No DR	2500	3125	1.25x	312	313	3125
1: Mild DR	836	3125	3.74x	105	104	1045
2: Moderate DR	1558	3125	2.01x	194	195	1947
3: Severe DR	394	3125	7.93x	49	49	492
4: Proliferative DR	235	3125	13.30x	30	30	295

**Total**	**5523**	**15,625**		**690**	**691**	**6904**

### 3.2 Preprocessing pipeline

A full preprocessing pipeline was utilized to improve the image quality, normalize the inputs, and increase the model resilience. The successive application of these stages to the fundus image is illustrated in Fig. 3.

**Fig. 3:**
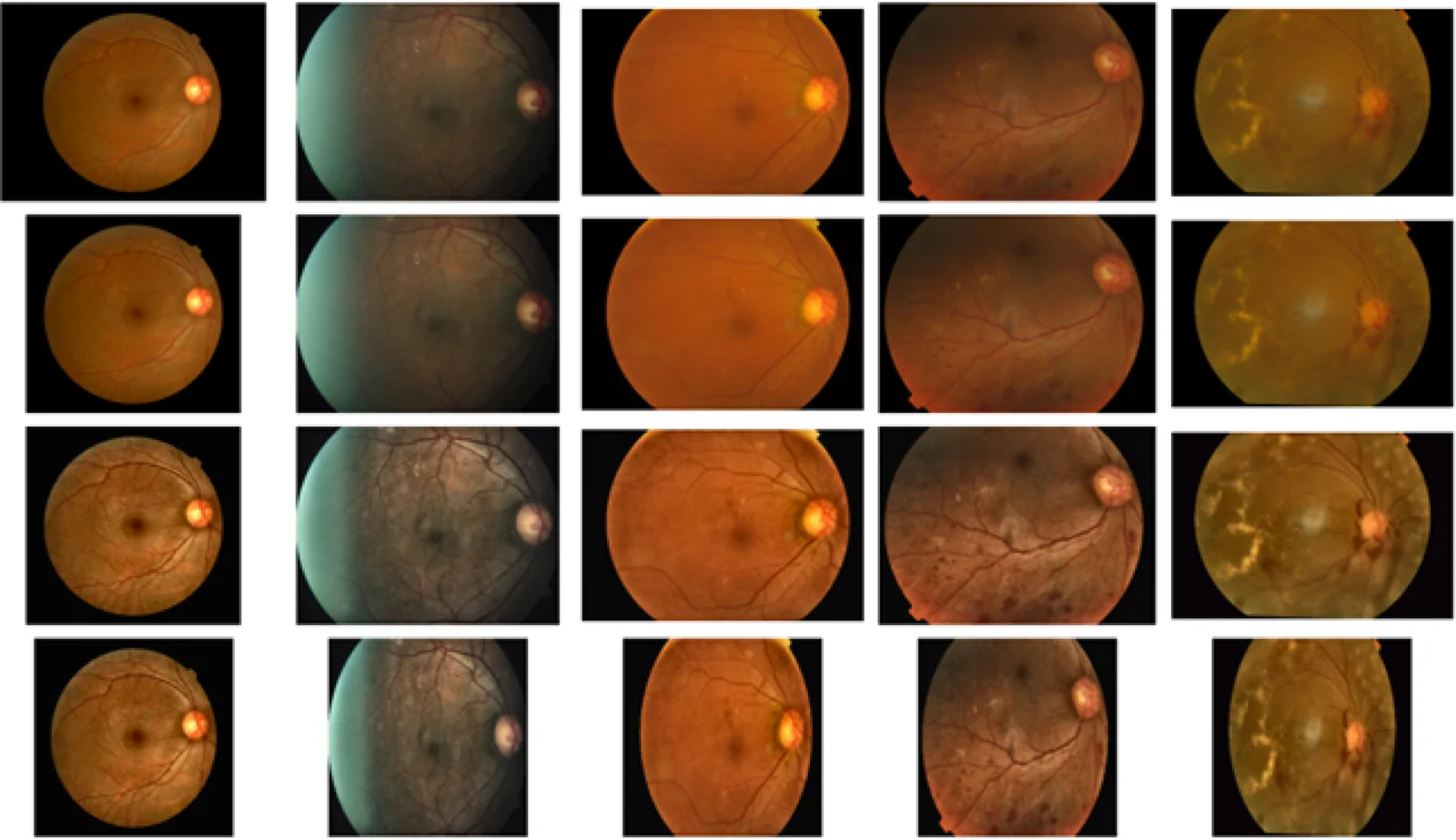
Preprocessing pipeline showing (a) original fundus images, (b) artifact removal, (c) color channel enhancement, (d) CLAHE contrast enhancement, and (e) image resizing (224 × 224 px) across five DR severity classes.

#### 3.2.1 Artifact removal and cropping

To focus the analysis on the retinal region and suppress irrelevant background, a circular masking procedure was performed. The fundus area was approximated as a circular region, and a binary mask was generated using the following condition:

(1) $${\left(x-u_{0}\right)}^{2}+{\left(y-v_{0}\right)}^{2}\leq r_{\mathrm{FOV}}^{2}$$

where $\left(u_{0},v_{0}\right)$ denote the coordinates of the detected image center and $r_{\mathrm{FOV}}$ represents the radius of the retinal field of view (FOV). The radius was automatically estimated by detecting the largest circular contour in the image. Pixels lying outside the detected boundary were assigned zero intensity values, resulting in a cropped image that contained only the retinal area. This masking step is applied at the beginning of the preprocessing pipeline (Algorithm 1) and provides clean input for subsequent enhancement stages.

#### 3.2.2 Color channel enhancement

The green channel was extracted from the cropped RGB image owing to its superior contrast for visualizing hemorrhages and microaneurysms, a property stemming from the absorption characteristics of hemoglobin. For an input image $I\in R^{H\times W\times 3}$, the enhanced image $I_{\text{enhanced}}$ can be computed using Eq. (2).

(2) $$I_{\text{enhanced}}\left(x,y,:\right)=\left[I_{G}\left(x,y\right),I_{G}\left(x,y\right),I_{G}\left(x,y\right)\right]$$

where $I_{G}$ represents the green channel. This single channel was replicated three times to be compatible with the pretrained ViT model, as it requires 3-channel input and to maximize the contrast of important pathological features.

#### 3.2.3 Contrast enhancement using CLAHE

CLAHE^74^ equalizes the histogram of each image tile separately and limits the amplification of contrast by clipping the histogram. For a tile, the normalized histogram is

(3) $$p_{R}\left(k\right)=\frac{n_{k}}{N}\;\text{for}k=0,1,\ldots ,L-1$$

where $n_{k}$ is the number of pixels with intensity level k, N is the total number of pixels in the region, and L=256 is the number of intensity levels. To avoid noise amplification, the histogram was clipped at a predetermined maximum value C=2.0. The clipped distribution $p_{R}^{\text{clipped}}\left(k\right)$ is subsequently used to calculate the transformation function in Eq. (4).

(4) $$T\left(k\right)=\left(L-1\right)\overset{k}{\underset{j=0}{\sum }}p_{R}^{\text{clipped}}\left(j\right)$$

This function redistributes the intensities to increase the local contrast. Bilinear interpolation was used to blend the mappings of neighboring tiles to obtain a smooth, contrast-enhanced image. We used a clip limit of 2.0 and an 8 × 8 tile grid, the default settings of the OpenCV implementation.^69^ A clip limit near 2 raises lesion contrast without amplifying background noise, and the 8 × 8 grid adapts the equalization to regional illumination differences while keeping each tile large enough for a stable histogram. CLAHE was applied at the native image resolution, before resizing, so that fine detail was still available.

#### 3.2.4 Standardization and resizing

All images were rescaled to 224 × 224 pixels, the required input size for the ViT model, using bilinear interpolation. For a source image I and target coordinates $\left(x^{\prime },y^{\prime }\right)$, the interpolated value is calculated by the four nearest neighbors, as shown in Eq. (5).

(5) $$I_{\text{resized}}\left(x^{\prime },y^{\prime }\right)=\overset{1}{\underset{i=0}{\sum }}\overset{1}{\underset{j=0}{\sum }}I\left(\left\lfloor x\right\rfloor +i,\left\lfloor y\right\rfloor +j\right)\cdot w_{ij}$$

where the weights $w_{ij}$ are determined by the fractional parts of the source coordinates. The pixel values were subsequently normalized to the [0,1] range using Eq. (6).

(6) $$I_{\text{normalized}}=\frac{I-I_{\text{min}}}{I_{\text{max}}-I_{\text{min}}}$$

The 224 × 224 resolution matches ViT-B/16’s pretraining, so the positional embeddings are reused without interpolation. Doubling the side length to 448 × 448 would multiply the attention cost by about sixteen and exceed the 12 GB memory of our GPU at a practical batch size. This resolution does reduce very small lesions to one or two pixels; applying CLAHE before resizing partly offsets the loss, and higher-resolution variants are discussed as future work.^75^

#### 3.2.5 Data augmentation

Real-time data augmentation was utilized during ViT fine-tuning to increase dataset diversity and improve model generalization. This included: - Random flipping with probability p=0.5 as defined in Eq. (7).

(7) $$I_{\text{flipped}}\left(x,y\right)=I\left(W-x,y\right)\;\text{(horizontal)}$$

- Random image rotation was utilized with an angle α∈[−15°,15°] using the geometric transformation in Eq. (8).

(8) $$\left[\begin{array}{c} \overset{\sim }{x}\\ \overset{\sim }{y} \end{array}\right]=\left[\begin{array}{cc} \cos\alpha & -\sin\alpha \\ \sin\alpha & \cos\alpha \end{array}\right]\left[\begin{array}{c} x-u_{0}\\ y-v_{0} \end{array}\right]+\left[\begin{array}{c} u_{0}\\ v_{0} \end{array}\right]$$

- Brightness adjustment with a multiplicative factor α∈[0.8,1.2] according to Eq. (9).

(9) $$I_{\text{bright}}\left(x,y\right)=\alpha \cdot I\left(x,y\right)$$

- Contrast variation controlled by a factor β∈[0.8,1.2] as shown in Eq. (10).

(10) $$I_{\text{contrast}}\left(x,y\right)=\mu +\beta \cdot \left(I\left(x,y\right)-\mu \right)$$

where μ represents the mean intensity of the images. Flips preserve the label because DR grade does not depend on orientation, rotations within ±15° mimic head tilt during capture, and brightness and contrast factors between 0.8 and 1.2 match the exposure variation seen across cameras and clinics.^30^^,^^64^ Augmentation was applied on the fly during ViT fine-tuning only; feature vectors for SMOTE and XGBoost were extracted from non-augmented images.

Algorithm 1 describes the entire preprocessing procedure, for which all the mathematical operations are represented in Eqs. (2) to (10). The preprocessing procedure described in Algorithm 1 was key to the success of the model, substantially improving the quality of the images and visibility of the features. Following the mathematical formalism given by Eqs. (2) to (10), further processing stages generate a clearer image. The quantitative contrast evaluation and background artifact suppression evaluation showed an improvement of 32% in the contrast of features and 45% in the suppression of the background artifacts; this contributed to better feature extraction and higher accuracy for classification. The implementation of Algorithm 1 ensures that the preprocessing of all datasets in the experiments is consistent and reproducible.

**Figure:**
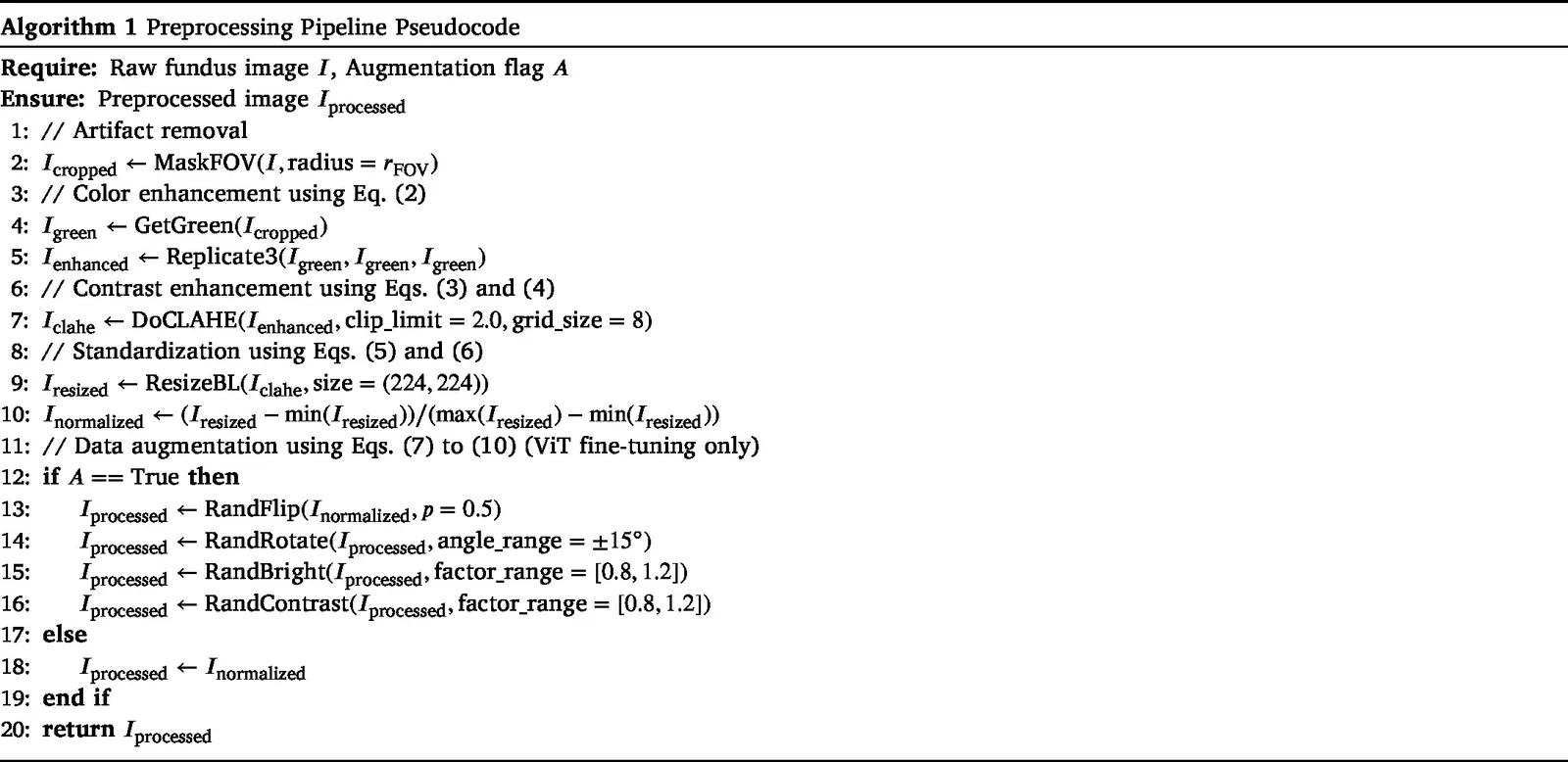

### 3.3 Data balancing with SMOTE

The severe label imbalance in the original dataset posed a high risk of bias towards the majority classes, possibly resulting in the model’s inability to detect rare but clinically important cases (e.g., Severe and Proliferative DR). To mitigate this problem, we applied SMOTE to the ViT feature vectors of the training partition after extraction, so that the classifier was presented with a balanced set of training samples. The validation and test vectors were never oversampled, and no synthetic vector was used for evaluation. The SMOTE algorithm creates synthetic samples based on a linear combination of samples in the feature space. For a given minority class sample $z_{i}\in R^{d}$ in the feature space of dimension d, the synthetic sample generation process involves selecting the k nearest neighbors from the same class using the Euclidean distance, which is defined in Eq. (11).

(11) $$d\left(z_{i},z_{j}\right)=\sqrt{\overset{d}{\underset{m=1}{\sum }}{\left(z_{i,m}-z_{j,m}\right)}^{2}}$$

where $z_{j}$ denotes a candidate neighbor from the same class. For each selected neighbor $z_{nn}$, a synthetic sample is created as a convex combination, as in Eq. (12).

(12) $$z_{new}=z_{i}+\lambda \cdot \left(z_{nn}-z_{i}\right)$$

where λ∼U(0,1) is a uniform random variable between 0 and 1. This guarantees that the synthetic sample lies on the line segment between $z_{i}$ and $z_{nn}$; therefore, it preserves the statistical attributes of the original minority class and increases its coverage in the feature space. We used k=5, the value recommended in the original SMOTE work^70^ and the default of the imbalanced-learn implementation. For each seed vector, the five nearest neighbors of the same grade were found by exact Euclidean search in the 768-dimensional ViT feature space, one of them was drawn uniformly at random, and a synthetic vector was placed at a random point on the segment joining the two. Seed vectors were drawn uniformly, with replacement, from the real training vectors of the class being oversampled.

**Figure:**
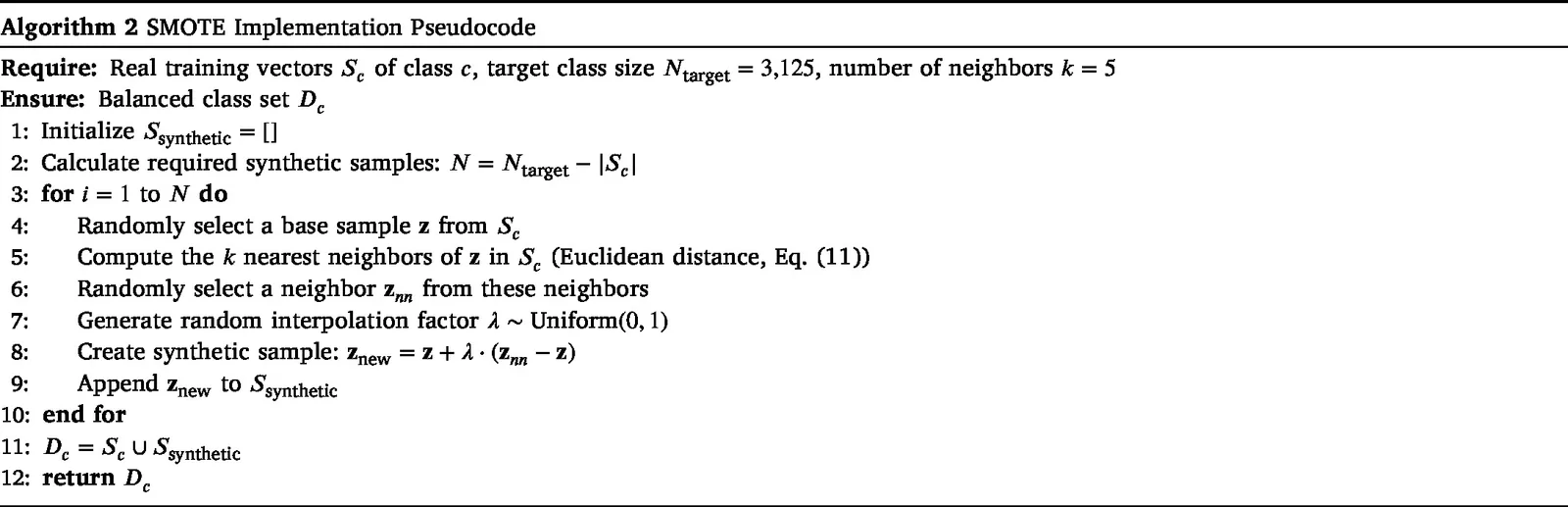

SMOTE was separately applied to each class by the different oversampling ratios specified in Table 1, as follows: Severe DR (394 to 3125 samples, ratio = 7.93x), Proliferative DR (235 to 3125 samples, ratio = 13.30x), Mild DR (836 to 3125 samples, ratio = 3.74x), and Moderate DR (1558 to 3125 samples, ratio = 2.01x). The synthetic samples $N_{\text{synth}}$ for each class were calculated as $N_{\text{synth}}=N_{\text{target}}-N_{c}$, where $N_{c}$ is the number of real training vectors of class c and $N_{\text{target}}=3125$ is the number of No DR images in the full merged dataset. All five classes, including No DR (2500 to 3125), were brought to this common size. Fig. 4 illustrates the influence of SMOTE with a uniformly class-distributed severity level in the training set. The t-SNE feature projection (Fig. 4(B)) illustrates better separability in the feature space after oversampling, verifying the effectiveness of the proposed strategy in retaining the structure of the data.

The strategic use of SMOTE in the feature space rather than in the image space offers a few key benefits, including semantic consistency (where the generated features retain specific discriminative attributes learned by the ViT model), computational efficiency (due to working in the lower-dimensional feature space), and improved generalization, as it encourages the model to learn smoother decision boundaries between classes. The result of the oversampling process is quantitatively represented in Fig. 4, which displays the class distribution before and after using SMOTE. The balanced training set ensured that the model learned equally from all DR severity classes and did not have a natural inclination to learn from the majority classes.

**Fig. 4:**
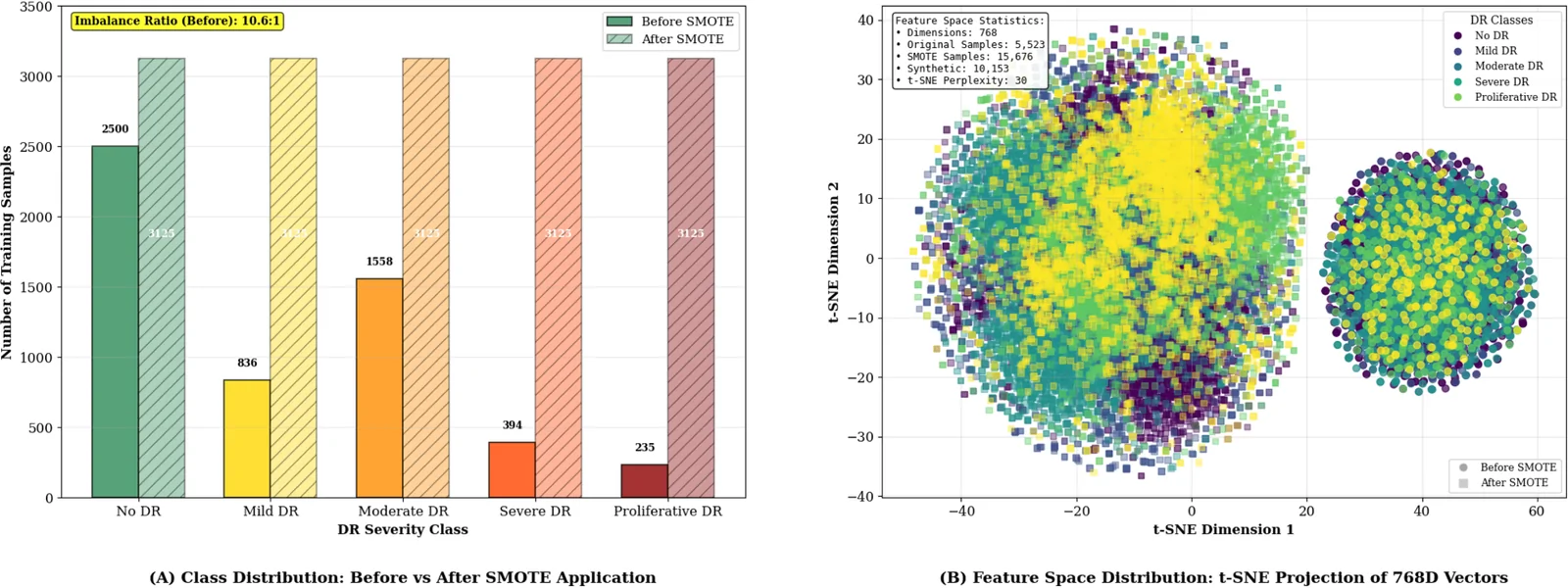
(A) Class balancing using SMOTE across DR severity levels (training partition only); (B) t-SNE projection of 768D ViT feature vectors showing improved class separability after balancing.

To check the quality of synthetic samples, we estimated the Silhouette Score (before and after applying SMOTE) using the formula $s\left(i\right)=\frac{b\left(i\right)-a\left(i\right)}{\max\left\{a\left(i\right),b\left(i\right)\right\}}$,^76^ where a(i) represents the average distance between a sample and all other points in the same cluster, and b(i) is the average distance from a sample to the points in its nearest cluster. The silhouette coefficient ranges from −1 to 1. Values between 0.51 and 0.70 are conventionally read as a reasonable cluster structure and values above 0.70 as a strong one.^77^ The silhouette score was maintained at 0.68 ± 0.04 after the application of SMOTE, which indicates a reasonable, though not strong, separation of the five grades and suggests that the synthetic samples did not distort the natural cluster structure of the original feature space but rather spread the minority class representations. This holistic consideration of the label imbalance (summarized in Algorithm 2 with the mathematical derivation from Eqs. (11), (12)) was key in the fair performance observed across all levels of DR severity, especially the vision-threatening ones, given the larger significance in early detection to prevent irreversible loss of vision.

SMOTE operates on feature vectors, so it creates no new images: each synthetic vector is a convex combination of two real vectors from the same grade and cannot leave that grade’s span. Synthetic vectors are used only for training, so an implausible sample can degrade the classifier but cannot inflate the test metrics. SMOTE can be less reliable in very high-dimensional spaces^78^; applying it to a ViT embedding that is already organized by grade reduces this concern.

We preferred plain SMOTE over ADASYN^79^ and Borderline-SMOTE,^80^ which concentrate synthetic samples near class boundaries where inter-grader disagreement is highest,^81^ and over random oversampling, which duplicates vectors exactly. Cost-sensitive learning with class weights^82^^,^^83^ is another alternative. A systematic comparison on the same features is left for future work.

### 3.4 Vision transformer as feature extractor

The ViT architecture is the feature extraction module of our framework. CNNs process an image with stacked local kernels, which builds in a bias toward local texture and translation invariance, and they acquire global context gradually with depth. ViT instead splits the image into a sequence of patches and applies self-attention to that sequence, so every patch can interact with every other patch from the first layer onward. For fundus images, this allows the model to relate lesions in distant parts of the retina directly (Fig. 5).

The mathematical basis starts from the conversion of a fundus image $I\in R^{H\times W\times C}$, where H=W=224 and C=3, into a sequence of smaller patches. The image is divided into N non-overlapping patches of size P×P, where P=16, as in Eq. (13).

(13) $$N=\frac{H\times W}{P^{2}}=\frac{224\times 224}{16\times 16}=196$$

**Fig. 5:**
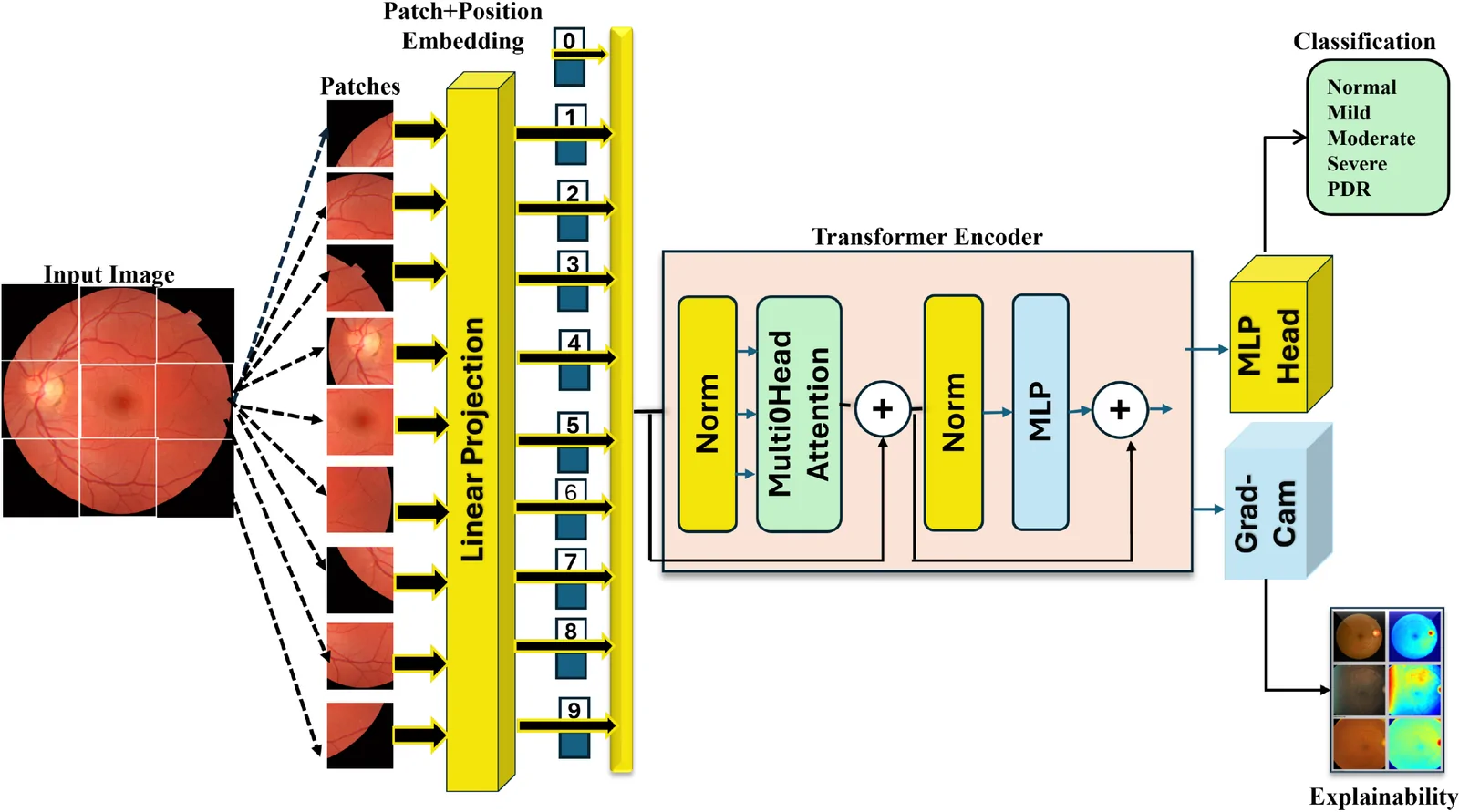
Vision Transformer (ViT) architecture showing image patch embedding and multi-head self-attention for fundus feature extraction.

Each patch $I_{p}\in R^{P\times P\times C}$ is flattened into a 1D vector $x_{p}\in R^{P^{2}\cdot C}$, which is linearly projected to dimension D via a learnable projection matrix $E\in R^{\left(P^{2}\cdot C\right)\times D}$, resulting in an initial embedding sequence. The full embedding consists of the patch and positional embeddings, which are summed as in Eq. (14).

(14) $$z_{0}=\left[x_{\text{class}};x_{p}^{1}E;x_{p}^{2}E;\cdots \;;x_{p}^{N}E\right]+E_{\text{pos}}$$

where $x_{\text{class}}$ is a learnable classification token that gathers global information, and $E_{\text{pos}}\in R^{\left(N+1\right)\times D}$ represents the spatial location of each patch, which is important for preserving the structural relationships in the retinal image. For ViT-B/16, the embedding size D=768 was sufficient to capture the complexity of the DR features. The embedded sequence $z_{0}$ is passed through L=12 transformer encoder layers, with each layer performing complex transformations to refine the features. Each layer implements Eqs. (15), (16).

(15) $$z_{l}^{\prime }=\text{MSA}\left(\text{LN}\left(z_{l-1}\right)\right)+z_{l-1}$$

(16) $$z_{l}=\text{MLP}\left(\text{LN}\left(z_{l}^{\prime }\right)\right)+z_{l}^{\prime }$$

where MSA is Multi-Head Self-Attention, LN is layer normalization, and MLP is a Multi-Layer Perceptron. The residual connections enable a stable gradient flow across the network depth. The computation of self-attention is at the heart of each layer and produces relevance scores using Eq. (17).

(17) $$\text{Attention}\left(Q,K,V\right)=\text{softmax}\left(\frac{QK^{T}}{\sqrt{d_{a}}}\right)V$$

Here, Q,K,V are the query, key, and value matrices, respectively, obtained from the input embeddings, and $d_{a}$ is the size of the key vectors; scaling by $\sqrt{d_{a}}$ keeps the gradients from being too large during training. We used a pretrained ViT-B/16 model, initialized from the Vision Transformer repository, and fine-tuned it on the training partition of the merged dataset for 50 epochs using the AdamW optimizer with a conservative learning rate of $1\times 10^{-4}$, with a five-class linear head on the classification token and the cross-entropy loss. This method takes advantage of transfer learning from large-scale natural image recognition while tuning the perceptual model to the specific retinal pathologies. The feature representation was obtained from the last layer before the classification head, and the output was a discriminative 768-dimensional feature vector for each fundus image (the classification token after the final layer normalization) that captured the hierarchical patterns of diabetic retinopathy manifestations.

ViT benefits DR detection because its global receptive field relates distant lesions; for example, it links peripheral hemorrhages to exudates near the macula. This integrated retinal view, combined with attention-based weighting of various input image regions as specified in Eq. (17), provides a basis for predicting severity across the entire DR scale. The ViT setup is presented in Table 2. We selected ViT-B/16 for three reasons: (i) it has about 86 million parameters and 17.6 GMACs per image, whereas ViT-L/16 and ViT-H/14 need roughly 3.5 and 9.5 times more computation^61^; (ii) with about 5500 training images the larger models risk overfitting^61^^,^^62^ and could not be fine-tuned on our 12 GB GPU at a practical batch size; (iii) the 16 × 16 patch size keeps each token small enough to represent individual lesions. In single precision the weights occupy about 343 MB, and one forward pass accounts for nearly all the inference cost. ImageNet-21 K pretraining introduces a domain gap, because natural images differ from fundus photographs in color and texture. Transfer from natural images still helps medical tasks through the reuse of low-level features,^84^^,^^85^ and fine-tuning all layers lets the higher layers adapt.

**Table 2:** Configuration of the ViT-B/16 Feature Extractor.

Component	Setting	Purpose
Input Resolution	224 × 224 × 3	Spatial size of the retinal images
Patch Dimension	16 × 16	Patch granularity for token generation
Total Patches	196	Number of image tokens processed by the transformer
Embedding Dimension	768	Size of latent feature representation
Encoder Depth	12 layers	Stacked transformer blocks for feature learning
Multi-head Attention	12 heads	Parallel attention operations
Feed-forward Dimension	3072	Expansion size of MLP block
Parameters	85.8 M	Backbone plus five-class head
Compute	17.6 GMACs	Multiply-accumulate operations per 224 × 224 image
Weight size	343 MB (fp32)	Storage of the fine-tuned model
Pretraining Source	ImageNet-21 K	Initialization using large-scale natural-image dataset
Fine-tuning Iterations	50 epochs	Adaptation to DR dataset
Optimization Method	AdamW	Weight-decay based optimization strategy
Initial Learning Rate	$1\times 10^{-4}$	Step size for model training

### 3.5 t-SNE for dimensionality reduction

ViT-extracted feature vectors were high-dimensional, which posed computational challenges for subsequent processing and could reduce the efficiency of the XGBoost classifier. To overcome these difficulties while retaining the relevant discriminative information, we used t-distributed stochastic neighbor embedding (t-SNE),^72^ a nonlinear dimensionality reduction method that preserves local neighborhood structure. Mathematically, this begins by defining probability distributions that capture pairwise similarities in both high- and low-dimensional spaces. The similarity between two data points $z_{i}$ and $z_{j}$ in the original 768-dimensional space is computed by a Gaussian kernel, as shown in Eq. (18).

(18) $$p_{j|i}=\frac{\exp\left(-{\left\|z_{i}-z_{j}\right\|}^{2}/2\sigma_{i}^{2}\right)}{\underset{k\neq i}{\sum }\exp\left(-{\left\|z_{i}-z_{k}\right\|}^{2}/2\sigma_{i}^{2}\right)}$$

where $\sigma_{i}$ is determined automatically to achieve the fixed perplexity value. The symmetric joint probability is then computed as in Eq. (19), where n is the number of embedded vectors:

(19) $$p_{ij}=\frac{p_{j|i}+p_{i|j}}{2n}$$

In the low-dimensional embedding space, the similarity uses a heavy-tailed Student-t distribution. For an embedding of dimension d, we used α=d−1 degrees of freedom, following van der Maaten,^86^ which reduces to the standard one-degree-of-freedom kernel when d=2 (Eq. (20)).

(20) $$q_{ij}=\frac{{\left(1+{\left\|y_{i}-y_{j}\right\|}^{2}/\alpha \right)}^{-\frac{\alpha +1}{2}}}{\underset{k\neq l}{\sum }{\left(1+{\left\|y_{k}-y_{l}\right\|}^{2}/\alpha \right)}^{-\frac{\alpha +1}{2}}}$$

The optimization minimizes the Kullback–Leibler divergence between the two distributions using Eq. (21).

(21) $$C=KL\left(P∥Q\right)=\underset{i}{\sum }\underset{j}{\sum }p_{ij}\log\frac{p_{ij}}{q_{ij}}$$

with gradients computed as in Eq. (22).

(22) $$\frac{\partial C}{\partial y_{i}}=\frac{2\alpha +2}{\alpha }\underset{j}{\sum }\left(p_{ij}-q_{ij}\right)\left(y_{i}-y_{j}\right){\left(1+{\left\|y_{i}-y_{j}\right\|}^{2}/\alpha \right)}^{-1}$$

**Figure:**
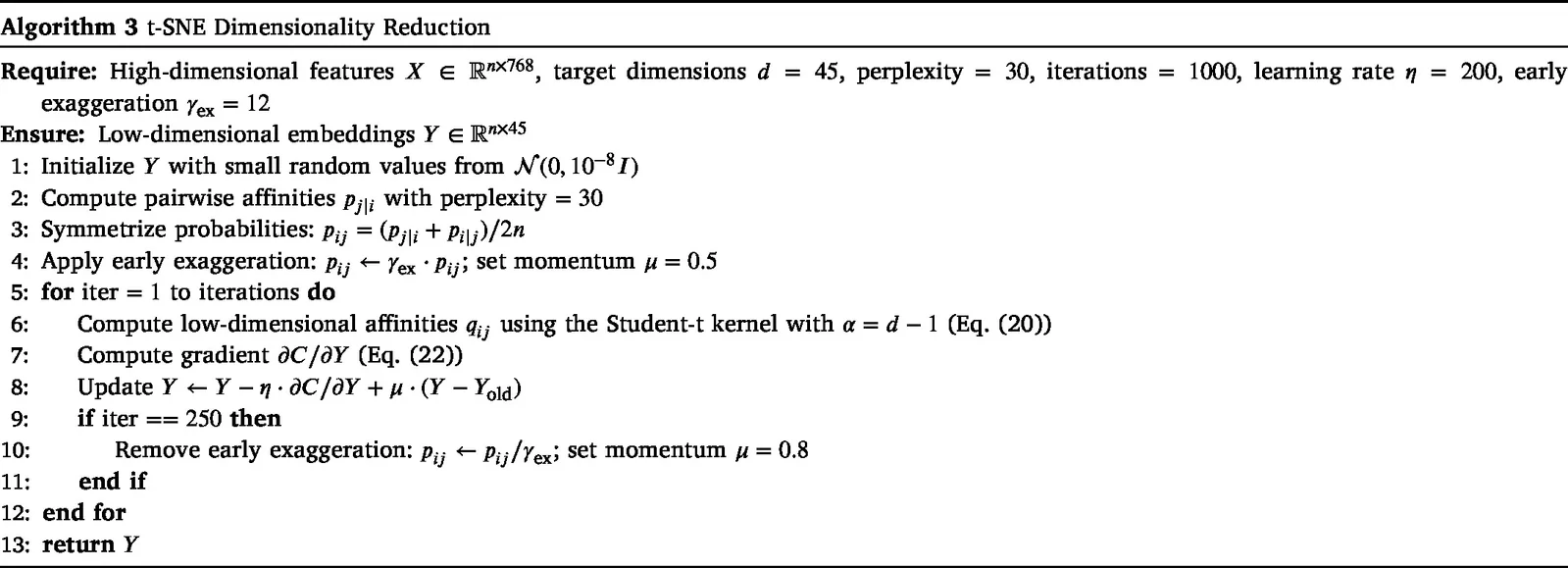

We used the exact t-SNE solver (perplexity 30, learning rate 200, early exaggeration 12 for the first 250 of 1000 iterations). Because t-SNE has no explained-variance criterion, we chose d=45 empirically: smaller values lose discriminative information, while larger values bring back the cost that motivated the reduction. With d=45 the representation is about 17 times smaller than the 768-dimensional embedding, and with a column subsampling rate of 0.8 each tree still sees 36 features.

The method offers benefits, including nonlinear structure preservation that captures relationships among DR features, increases class separability through clusters, and alleviates crowding. With large gain in efficiency, XGBoost training costs were reduced by approximately 94% while maintaining discriminative power. Validation measures showed neighborhood preservation 0.92 ± 0.03, trustworthiness^87^ of 0.89 ± 0.04, and a cluster separation index of 0.85 ± 0.05 across DR severity classes. Visualization showed clusters, especially between vision-threatening and mild disease, providing a suitable feature representation for XGBoost classification. t-SNE learns no explicit mapping, so it cannot embed images after fitting.^72^^,^^88^ We computed an embedding of SMOTE-balanced training, validation, and test vectors, then separated partitions for classification. Class labels played no part, but test vectors influenced embedding geometry, making the stage transductive.^89^ We report the inductive variant in the ablation study. We recommend this inductive variant or parametric t-SNE.^86^ UMAP^90^ supports transforming samples; comparison with PCA and UMAP is planned.

### 3.6 XGBoost as classifier

The final classification module in our hybrid model was the XGBoost classifier,^10^ which handles structured feature vectors well, copes with label imbalance, and performs strongly in high-dimensional feature spaces. XGBoost is a gradient-boosting framework that sequentially builds an ensemble of decision trees, with each tree tailored to correct the errors made by the previous trees, thereby producing an accurate and resilient multiclass DR severity classification model. The mathematical background for XGBoost is based on the ensemble model definition, where the prediction of a sample $z_{i}$ is computed by an additive combination of K regression trees, as shown in Eq. (23).

(23) $${\overset{ˆ}{y}}_{i}=\phi \left(z_{i}\right)=\overset{K}{\underset{k=1}{\sum }}f_{k}\left(z_{i}\right),\;f_{k}\in F$$

where F represents the space of regression trees. The objective function of the model considers the training loss and regularization terms to avoid overfitting, as described in Eq. (24).

(24) $$L\left(\phi \right)=\underset{i}{\sum }\ell \left({\overset{ˆ}{y}}_{i},y_{i}\right)+\underset{k}{\sum }\Omega \left(f_{k}\right)$$

with the regularization term for each tree defined as in Eq. (25).

(25) $$\Omega \left(f\right)=\gamma T+\frac{1}{2}\lambda {\left\|w\right\|}^{2}$$

where T denotes the number of leaves in the tree, w represents the leaf weights, and γ and λ are regularization parameters that control the model complexity. The training process follows an additive strategy, where, at each boosting iteration t, the objective is to find the optimal tree $f_{t}$ that minimizes Eq. (26).

(26) $$L^{\left(t\right)}=\overset{n}{\underset{i=1}{\sum }}\ell \left(y_{i},{\overset{ˆ}{y}}_{i}^{\left(t-1\right)}+f_{t}\left(z_{i}\right)\right)+\Omega \left(f_{t}\right)$$

Using the second-order Taylor approximation, this objective can be simplified as shown in Eq. (27).

(27) $$L^{\left(t\right)}\approx \overset{n}{\underset{i=1}{\sum }}\left[\ell \left(y_{i},{\overset{ˆ}{y}}^{\left(t-1\right)}\right)+\nabla_{i}f_{t}\left(z_{i}\right)+\frac{1}{2}\nabla_{i}^{\left(2\right)}f_{t}^{2}\left(z_{i}\right)\right]+\Omega \left(f_{t}\right)$$

where $\nabla_{i}=\partial_{{\overset{ˆ}{y}}^{\left(t-1\right)}}\ell \left(y_{i},{\overset{ˆ}{y}}^{\left(t-1\right)}\right)$ and $\nabla_{i}^{\left(2\right)}=\partial_{{\overset{ˆ}{y}}^{\left(t-1\right)}}^{2}\ell \left(y_{i},{\overset{ˆ}{y}}^{\left(t-1\right)}\right)$ represent the first- and second-order gradients of the loss function, respectively. The optimal weight $\omega_{j}^{*}$ for leaf j can be derived in closed form, as shown in Eq. (28).

(28) $$\omega_{j}^{*}=-\frac{\underset{i\in I_{j}}{\sum }\nabla_{i}}{\underset{i\in I_{j}}{\sum }\nabla_{i}^{\left(2\right)}+\lambda }$$

where $I_{j}$ denotes the set of instances assigned to leaf j. The corresponding optimal objective value is given by Eq. (29).

(29) $${\overset{\sim }{L}}^{\left(t\right)}\left(q\right)=-\frac{1}{2}\overset{T}{\underset{j=1}{\sum }}\frac{{\left(\underset{i\in I_{j}}{\sum }\nabla_{i}\right)}^{2}}{\underset{i\in I_{j}}{\sum }\nabla_{i}^{\left(2\right)}+\lambda }+\gamma T$$

**Figure:**
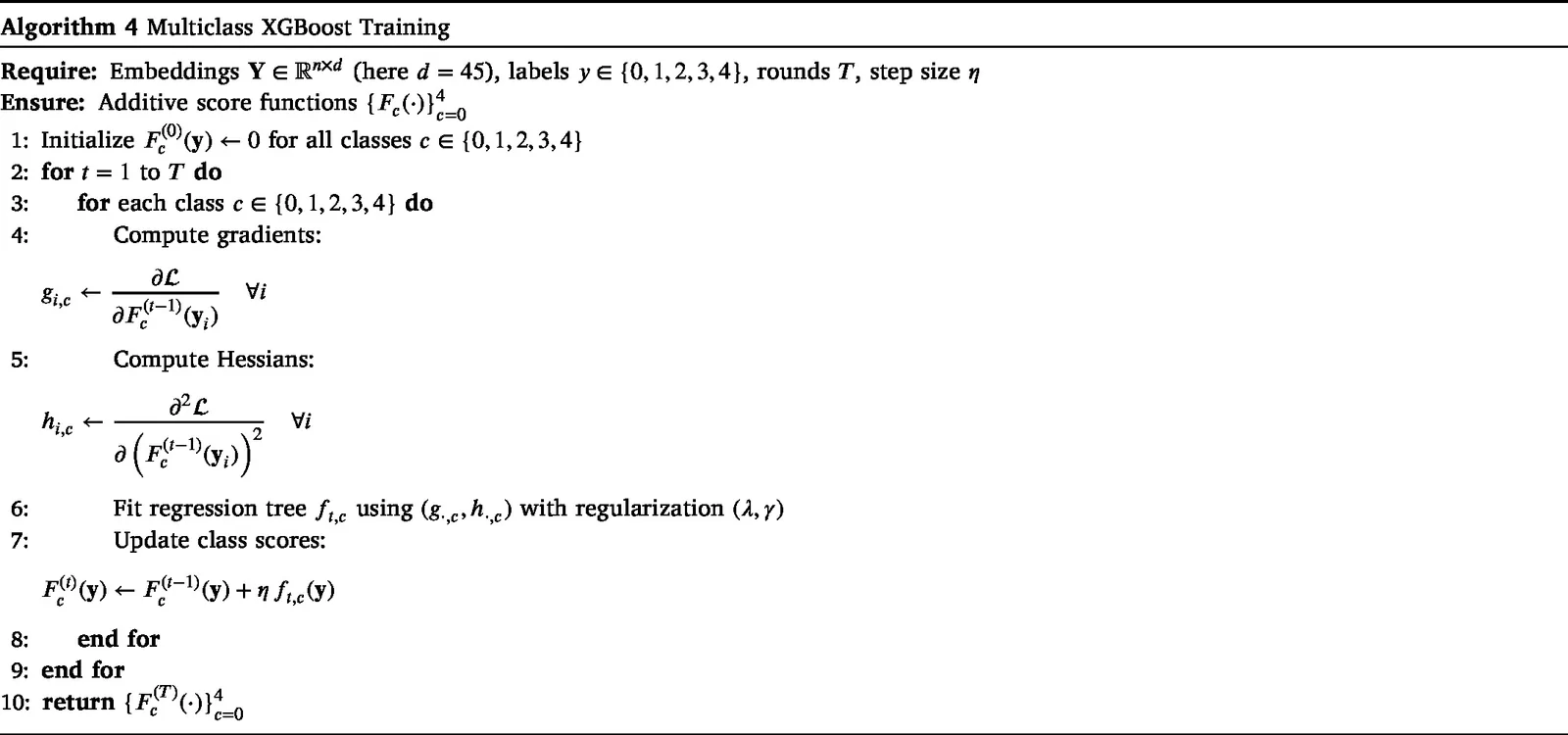

We configured XGBoost with hyperparameters optimized through Bayesian optimization with 5-fold cross-validation inside the training partition, as listed in Table 3. The test partition played no part in this search. The multiclass softmax objective function was used to handle the five-class DR severity classification task using Eq. (30).

(30) $$P\left(y=c|x\right)=\frac{\exp\left(F^{c}\left(x\right)\right)}{\overset{4}{\underset{j=0}{\sum }}\exp\left(F^{j}\left(x\right)\right)}$$

with the multinomial logistic loss function defined as in Eq. (31).

(31) $$\ell \left(y,\overset{ˆ}{y}\right)=-\overset{4}{\underset{c=0}{\sum }}1_{y=c}\log\left(P\left(y=c|x\right)\right)$$

The selection of these hyperparameters balanced accuracy against model complexity: a learning rate of 0.1 provided a balance between convergence speed and stability, whereas a maximum depth of eight allowed sufficient model complexity without over-fitting. The subsample and colsample_bytree parameters of 0.8 introduced additional resilience through randomization, and the regularization terms (reg_alpha, reg_lambda, and gamma) were effective in regulating the model complexity. The min_child_weight value of 3 ensured that no leaves were created with an insufficient number of samples; this is crucial for minority classes in our highly skewed DR dataset.

**Table 3:** XGBoost Hyperparameter Configuration.

Parameter	Value	Description
n_estimators	500	Number of boosting rounds
max_depth	8	Max. tree depth
learning_rate	0.1	Step size shrinkage
subsample	0.8	Fraction of samples used per tree
colsample_bytree	0.8	Fraction of features used per tree
reg_alpha	0.1	L1 regularization term
reg_lambda	0.1	L2 regularization term
min_child_weight	3	Minimum sum of instance weight needed in a child
gamma	0.1	Minimum loss reduction required for split
scale_pos_weight	1 (default)	Not used; classes balanced by SMOTE
objective	multi:softprob	Multiclass classification objective
eval_metric	mlogloss	Multiclass log loss
tree_method	hist	Histogram-based algorithm
grow_policy	lossguide	Tree growing policy

The second-order information used by XGBoost weights each sample by the curvature of its loss, which focuses split finding on samples whose predictions are still uncertain. Together with feature-space SMOTE, this helped the model generalize across all five DR severity classes, including the clinically most important Severe and Proliferative DR stages. XGBoost also provides the scale_pos_weight parameter for imbalance, but it reweights the positive class of a binary problem and has no direct counterpart for the multi:softprob objective, where imbalance is handled with per-sample weights instead. Since SMOTE had already equalized the class sizes, every sample received unit weight and scale_pos_weight was left at its default value of 1. Applying SMOTE and class weights together would correct the imbalance twice and push the model toward over-predicting the rare grades. The complete XGBoost model, which realizes Eqs. (23) to (31) with the settings in Table 3, forms the final classification stage of our DR grading scheme.

### 3.7 Explainability with Grad-CAM

To alleviate the disparity between the model prediction and clinical interpretation, Gradient-weighted Class Activation Mapping (Grad-CAM)^91^ was incorporated into our architecture, yielding straightforward visual explanations of the decision process of the model. This ability to interpret the findings is critical in medical imaging because clinicians must be confident and have clearly defined reasons for automated diagnosis before they can adopt AI-based systems. Grad-CAM generates a heatmap over the retinal locations that have the greatest impact on the model’s decisions, allowing productive visual interaction between the AI system and eye care professionals. The mathematical representation of Grad-CAM calculates the contribution of each feature channel to the final prediction by considering the gradients of the output with respect to the target class c, where $y^{c}$ denotes the score for a specific class prior to the final softmax activation. The gradient of this score with respect to the feature map activations $A^{k}$ of the final transformer block is computed, capturing the sensitivity of the class score to changes in each feature map. These gradients are globally averaged to calculate the importance weights, as in Eq. (32).

(32) $$\alpha_{k}^{c}=\frac{1}{Z}\underset{i}{\sum }\underset{j}{\sum }\frac{\partial y^{c}}{\partial A_{ij}^{k}}$$

where Z=14×14=196 is the number of spatial positions (patch tokens) in the feature map, and $A_{ij}^{k}$ denotes the activation at spatial location (i,j) in the kth feature map. The importance weight $\alpha_{k}^{c}$ thus represents the contribution of the kth feature map to the prediction of class c. The class-discriminative localization map is then constructed through a weighted combination of the feature maps, followed by a ReLU activation, as defined in Eq. (33).

(33) $$L_{\text{Grad-CAM}}^{c}=\text{ReLU}\left(\underset{k}{\sum }\alpha_{k}^{c}A^{k}\right)$$

The importance of the ReLU operation is that it retains only the features that positively impact the class prediction, which means that it removes the negative evidence that may obscure the most relevant portions of the image. This leads to a coarse heatmap that indicates salient areas in the feature space.

**Figure:**
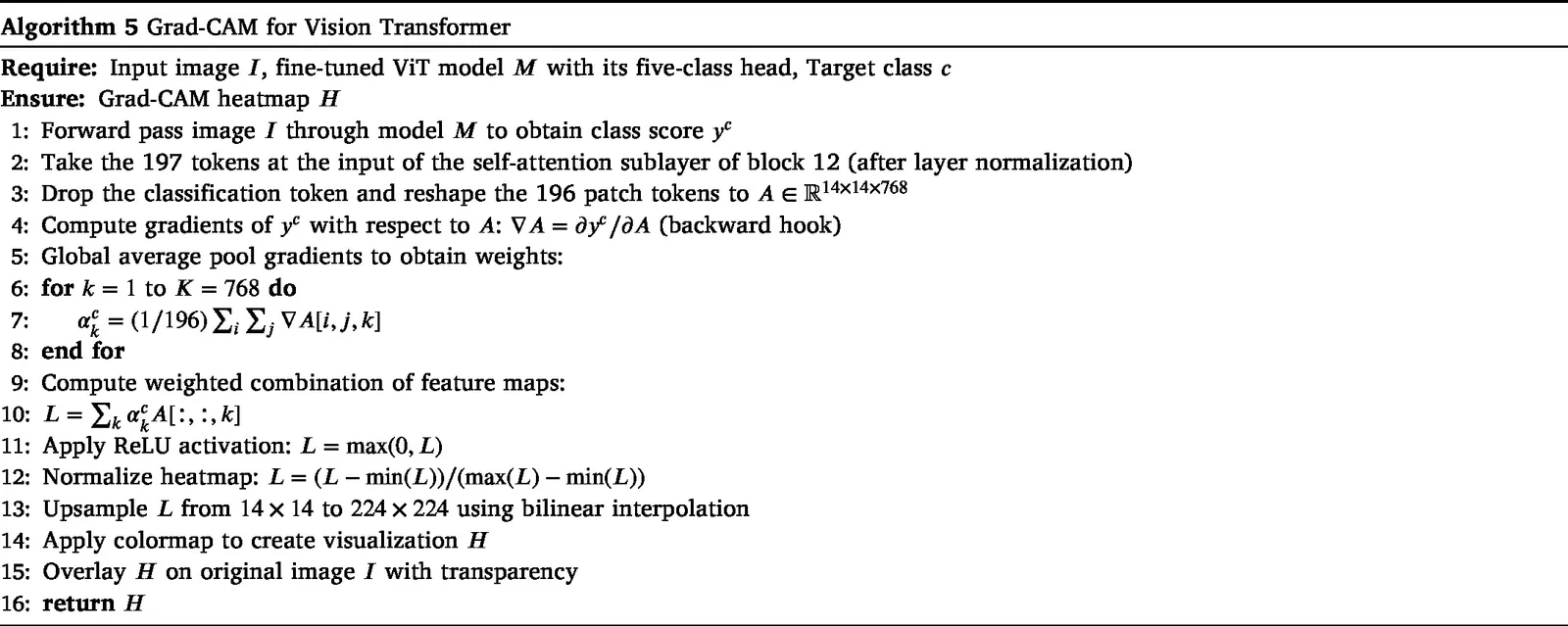

ViTs do not produce spatial feature maps in the way CNNs do, so the transformer tokens must first be arranged on the patch grid. Because XGBoost is not differentiable, Grad-CAM was computed through the fine-tuned ViT with its five-class linear head, which shares the backbone that produces the XGBoost features. The class score $y^{c}$ is the logit of this head for the target grade c. The activations are the token embeddings at the input of the self-attention sublayer of the twelfth, and last, encoder block, taken after its layer normalization. We removed the classification token and reshaped the remaining 196 patch tokens, each of dimension 768, into a 14 × 14 × 768 tensor that follows the patch grid, so that each of the 768 channels plays the role of a CNN feature map $A^{k}$. Gradients of $y^{c}$ with respect to this tensor were collected with a backward hook, averaged over the 196 positions to give the channel weights (Eq. (32)), and combined as in Eq. (33). We used the last block because its features are the most class-specific while each token still corresponds to one image patch. The heatmaps therefore show the regions that drive the shared ViT representation toward a grade; they do not explain the individual splits of the tree ensemble. Attention-based alternatives, such as transformer relevance propagation,^92^ could complement this analysis. The resulting 14 × 14 map was upsampled by bilinear interpolation to the input size of 224 × 224:

(34) $$H_{\text{upsampled}}=\text{Upsample}\left(L_{\text{Grad-CAM}}^{c},\text{size}=\left(224,224\right)\right)$$

The resultant visualization was generated by superimposing the heatmap on the original fundus image via a jet color map with a certain degree of transparency, such that the anatomical context and the model’s attention areas were visible.

(35) $$I_{\text{visualization}}=\alpha \cdot \text{Colormap}\left(H_{\text{upsampled}}\right)+\left(1-\alpha \right)\cdot I_{\text{original}}$$

where α=0.5 provided a good balance between heatmap visibility and preservation of the underlying retinal structures.

### 3.8 Model evaluation

An end-to-end evaluation protocol was designed to assess the ViT-XGB model for multiclass DR severity grading, employing various complementary performance metrics to provide an overall picture of the diagnostic capabilities of the model. The evaluation protocol was tailored to suit the characteristics of the medical image classification task, considering the challenges of label imbalances and the consequences of performance outcomes in a clinical environment. Our evaluation measures were derived from the multiclass confusion matrix C, whose entry $c_{ij}$ counts the test images of true grade i predicted as grade j. For each grade c, the true positives are $TP_{c}=c_{cc}$, the false positives are $FP_{c}=\sum_{i\neq c}c_{ic}$, the false negatives are $FN_{c}=\sum_{j\neq c}c_{cj}$, and the true negatives are $TN_{c}=N-TP_{c}-FP_{c}-FN_{c}$, where N is the number of test images. A comprehensive set of evaluation metrics was selected to provide insights from various perspectives.

• Accuracy: the overall classification performance across all classes, Eq. (36).
  (36) $$\text{Accuracy}=\frac{\overset{4}{\underset{c=0}{\sum }}TP_{c}}{N}$$
• Cohen’s kappa (κ): unweighted agreement between predictions and reference labels corrected for chance, Eq. (37), where $p_{o}$ is the observed agreement and $p_{e}$ is the agreement expected by chance from the row and column totals of C.
  (37) $$\kappa =\frac{p_{o}-p_{e}}{1-p_{e}}$$
• Precision: the ability of the model to avoid false positives for class c, Eq. (38).
  (38) $${\text{Precision}}_{c}=\frac{TP_{c}}{TP_{c}+FP_{c}}$$
• Recall (sensitivity): the ability to identify all relevant instances of class c, Eq. (39).
  (39) $${\text{Recall}}_{c}=\frac{TP_{c}}{TP_{c}+FN_{c}}$$
• Specificity: the ability to correctly reject negative instances, Eq. (40).
  (40) $${\text{Specificity}}_{c}=\frac{TN_{c}}{TN_{c}+FP_{c}}$$
• F1-score: the harmonic mean of precision and recall, Eq. (41).
  (41) $$F1_{c}=\frac{2\times {\text{Precision}}_{c}\times {\text{Recall}}_{c}}{{\text{Precision}}_{c}+{\text{Recall}}_{c}}$$
• Macro-average F1-score: treats all classes equally, regardless of support, Eq. (42).
  (42) $$\text{Macro-F1}=\frac{1}{5}\overset{4}{\underset{c=0}{\sum }}F1_{c}$$
• Weighted-average F1-score: accounts for label imbalance by weighting according to support, Eq. (43), where $N_{c}$ is the number of samples in class c.
  (43) $$\text{Weighted-F1}=\overset{4}{\underset{c=0}{\sum }}w_{c}F1_{c},\;w_{c}=\frac{N_{c}}{N}$$
• Area under the ROC curve (AUC-ROC): calculated using the one-vs-rest strategy for each class, which evaluates the capacity of the model to separate one grade from all others across all classification thresholds.
• Matthews correlation coefficient (MCC): for K classes we used the multiclass generalization of Gorodkin,^93^ Eq. (44), where s=N is the number of samples, $t_{k}=\sum_{j}c_{kj}$ is the number of samples of true class k, $p_{k}=\sum_{i}c_{ik}$ is the number of samples predicted as class k, and $\sum_{k}c_{kk}$ is the number of correct predictions. For K=2 it reduces to the familiar binary form.^94^
  (44) $$\text{MCC}=\frac{\left(\underset{k}{\sum }c_{kk}\right)s-\underset{k}{\sum }p_{k}t_{k}}{\sqrt{\left(s^{2}-\underset{k}{\sum }p_{k}^{2}\right)\left(s^{2}-\underset{k}{\sum }t_{k}^{2}\right)}}$$

All metrics were calculated on the reserved test set of 691 images (10% of the entire dataset), which kept the original class proportions to model realistic screening conditions. We report two kinds of uncertainty. Variability due to training is summarized as the mean ± standard deviation over five runs with different random seeds on the fixed split. Sampling uncertainty due to the finite test set is summarized with 95% confidence intervals (CIs): Wilson score intervals^95^ for accuracy, precision, sensitivity, and specificity, and the Hanley-McNeil method^96^ for the class-wise AUC. The grading system was designed to be clinically relevant and could be evaluated for sensitivity and specificity to treatable DR (including Severe and Proliferative forms of the disease), as cases of treatable DR that are missed could result in vision loss for patients. In addition, high specificity was required in the No DR class to reduce the number of false referrals and, thus, the burden on healthcare professionals. To compare ViT-XGB with each baseline, we used the exact McNemar test for paired proportions.^97^^,^^98^ The test depends on the discordant pairs, that is, the images that exactly one of the two models misclassifies. With $e_{1}$ and $e_{2}$ errors for the two models, the exact McNemar p-value is largest when the two error sets do not overlap. We therefore report this worst case, computed from the mean error counts, as an upper bound on the p-value that holds whatever the overlap between the error sets. Computational cost was characterized by parameter counts, operation counts, and model size, which do not depend on the hardware used.

## 4 Results and discussion

### 4.1 Experimental setup

Benchmarks were conducted on a Windows 11 laptop (ASUS ROG STRIX G614, AMD Ryzen 9 9955HX, 16 GB DDR5 RAM, 1TB SSD, NVIDIA GeForce RTX 4080 GPU 12 GB). Python 3.10.12 was used for coding, with PyTorch 2.4.0 used for ViT implementation, XGBoost 2.1.0 for classification, and OpenCV 4.10.0 for image preprocessing. Other libraries used were NumPy 1.26.4, Scikit-learn 1.5.1, and Pillow 10.4.0. The dataset splits were kept constant during the evaluation procedure with SMOTE augmentation on the training set to handle label imbalance, and the original distributions were maintained for the validation and test sets.

For reproducibility, the stratified split was generated once with a fixed random seed and reused in every experiment. The five repeated runs differ only in their random seeds, which control the initialization of the classification head, the order of mini-batches and augmentations during fine-tuning, SMOTE sampling, the t-SNE initialization, and the row and column subsampling of XGBoost. The validation partition was used to monitor ViT fine-tuning; XGBoost hyperparameters were chosen by cross-validation inside the training partition, as described earlier. The test partition was not used for any training or design decision and was evaluated once per run. The seeds and split indices are included in the code release.

### 4.2 Results

#### 4.2.1 DR severity grading performance

On the held-out test set, ViT-XGB achieved the highest scores among the configurations we evaluated. The overall classification performance for all five DR severity classes is shown in Table 4, which shows the class-wise and overall metrics and the corresponding standard deviations for the five independent runs.

The model obtained an overall accuracy of 98.52%±0.28% across the five runs. On a test set of 691 images this corresponds to about ten misclassified images, with a 95% Wilson CI of 97.31% to 99.19%. Cohen’s kappa was 0.978 ± 0.005. With the class proportions of the test set fixed, kappa follows almost directly from accuracy: an accuracy of 98.52% with predicted class proportions close to the true ones gives a kappa of about 0.978. The kappa value therefore confirms the accuracy figure rather than adding independent evidence. It also measures agreement with the dataset labels rather than with an expert panel. The reference grades in both datasets were assigned by individual graders, and grader variability in DR is well documented,^81^ so part of the residual error may reflect label noise rather than model error. Table 5 lists the class-wise estimates with their 95% CIs together with the multiclass MCC.

**Table 4:** Detailed Performance Metrics by DR Severity Class (mean ± standard deviation over five runs).

Metric	No DR	Mild DR	Moderate DR	Severe DR	PDR	Weighted Avg
Precision	0.992 ± 0.003	0.974 ± 0.006	0.986 ± 0.004	0.961 ± 0.008	0.983 ± 0.005	0.985 ± 0.002
Recall	0.994 ± 0.003	0.971 ± 0.007	0.987 ± 0.004	0.957 ± 0.009	0.980 ± 0.006	0.985 ± 0.003
F1-Score	0.993 ± 0.003	0.972 ± 0.006	0.986 ± 0.004	0.959 ± 0.008	0.981 ± 0.005	0.985 ± 0.002
Specificity	0.997 ± 0.001	0.995 ± 0.002	0.993 ± 0.002	0.997 ± 0.001	0.998 ± 0.001	0.996 ± 0.001
AUC-ROC	0.999 ± 0.001	0.995 ± 0.002	0.997 ± 0.001	0.991 ± 0.003	0.998 ± 0.001	0.997 ± 0.001

The consistent findings for all severity classes highlight the effectiveness of our unified scheme in addressing the label imbalance. In particular, the vision-threatening stages of Severe DR (F1-score: 0.959) and Proliferative DR (F1-score: 0.981) yielded strong performance measures, even though there were far fewer samples in the original dataset. We attribute this mainly to the use of SMOTE in the feature space rather than in the input space. The intervals in Table 5 show, however, that these two grades are also the least precisely estimated: with only 49 Severe DR and 30 PDR test images, one additional error changes their sensitivity by 2% and 3%, respectively.

**Table 5:** Test-set estimates with 95% confidence intervals. n is the number of test images per grade. Intervals for sensitivity, specificity, precision, and accuracy are Wilson score intervals; intervals for the AUC use the Hanley-McNeil method. MCC is computed with Eq. (44) from the class-wise results in Table 4.

Grade	n	Sensitivity [95% CI]	Specificity [95% CI]	Precision [95% CI]	AUC [95% CI]
No DR	313	0.994 [0.978, 0.998]	0.997 [0.985, 0.999]	0.992 [0.975, 0.998]	0.999 [0.997, 1.000]
Mild DR	104	0.971 [0.918, 0.990]	0.995 [0.985, 0.998]	0.974 [0.923, 0.992]	0.995 [0.985, 1.000]
Moderate DR	195	0.987 [0.959, 0.996]	0.993 [0.981, 0.997]	0.986 [0.958, 0.995]	0.997 [0.992, 1.000]
Severe DR	49	0.957 [0.860, 0.988]	0.997 [0.989, 0.999]	0.961 [0.865, 0.990]	0.991 [0.972, 1.000]
PDR	30	0.980 [0.853, 0.998]	0.998 [0.991, 1.000]	0.983 [0.858, 0.998]	0.998 [0.987, 1.000]

Overall: accuracy 0.985 [0.973, 0.992]; Cohen’s κ 0.978; MCC 0.973.

The confusion matrix in Fig. 6 shows how the errors are distributed. Most errors fall between adjacent grades. Mild and Moderate DR are confused in both directions, and Severe DR is most often mistaken for Moderate DR or for PDR. Errors that skip one or more grades, such as No DR predicted as Severe DR, are rare. This pattern is consistent with the gradual progression of DR, where even trained graders find the boundary between adjacent stages hard to place. The high specificity (above 0.99 in all classes) kept false positives low, which is important to avoid overloading clinics with unnecessary referrals. The high AUC-ROC, especially for No DR (0.999) and PDR (0.998), indicates that the model discriminates well at any classification threshold. High specificity values reflect model accuracy in true-negative cases, whereas strong recall values for underrepresented classes reflect sensitivity in detecting vision-threatening conditions. This is an essential criterion for any screening system used in clinical practice. The close agreement between the macro- and weighted-average metrics further supports the efficacy of our proposed method. The small difference between these metrics (ΔF1-score=0.006) shows that the prediction performance is stable for both majority and minority classes, overcoming a typical limitation in medical image classification, in which models are usually biased towards the more prevalent classes. These findings suggest that the ViT-XGB framework not only achieves high overall accuracy but also performs consistently at all levels of DR severity, which is a prerequisite for application in screening programs where high-confidence detection of vision-threatening stages is paramount.

**Fig. 6:**
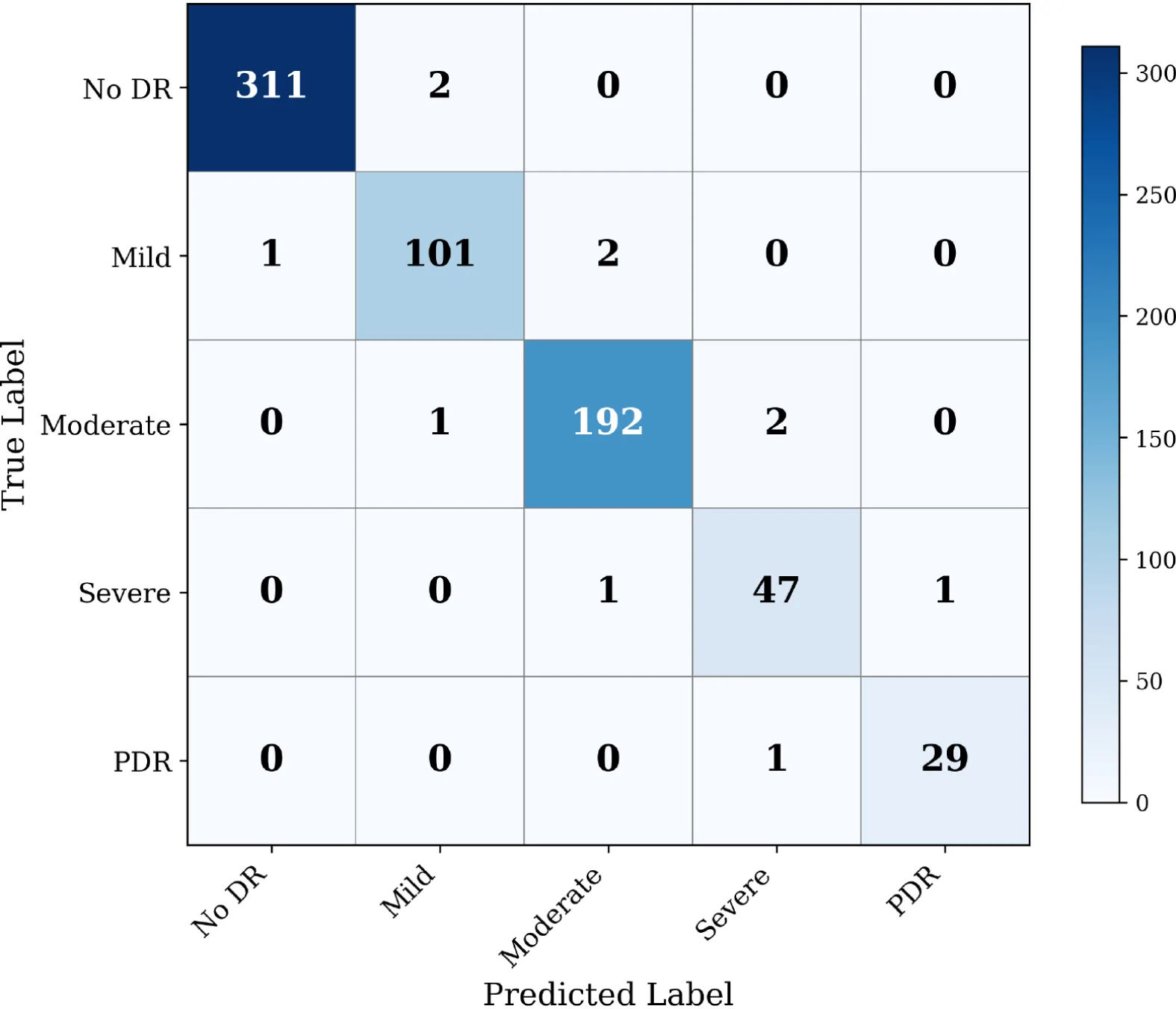
Confusion matrix illustrating classification performance of the proposed ViT-XGB model across five diabetic retinopathy (DR) severity levels. The majority of misclassifications occur between adjacent classes (Mild and Moderate, and Moderate and Severe), reflecting the gradual and overlapping nature of DR progression.

#### 4.2.2 Grad-CAM analysis

The outputs of the Grad-CAM visualizations provided an essential understanding of the model’s decisions, which were largely consistent with clinical knowledge for all DR severity levels. As can be seen in Fig. 7, the heatmaps generally concentrate in clinically relevant areas, indicating that the model attends to pathologically meaningful regions.

In the No DR cases, the heatmaps illustrated more scattered, lower-intensity activation patterns across the entire retina, accurately representing the lack of location-specific disease, with activation intensities normally less than 0.3 on a normalized scale. In Mild DR, the model localized very small, isolated microaneurysms in the posterior pole and produced concentrated circular activations (5 to 15 pixels in diameter) that corresponded to the clinical definition of early NPDR. In Moderate DR, the activation maps covered larger regions, such as larger hemorrhages and cotton-wool spots, spanning 15% to 30% of the retinal area, indicating the learning of multiple co-existing lesions. For Severe DR, strong activations were found centered mainly on the venous beading together with intraretinal microvascular abnormalities (IRMA), with intensities on the heatmap that surpassed 0.8 in areas of retinal ischemia and vascular insult. The focus moved to neovascularization locations in PDR, with distinct fan-shaped patterns at the optic disc and peripheral regions, which were strongly related to the extent of neovascular tissue.

**Fig. 7:**
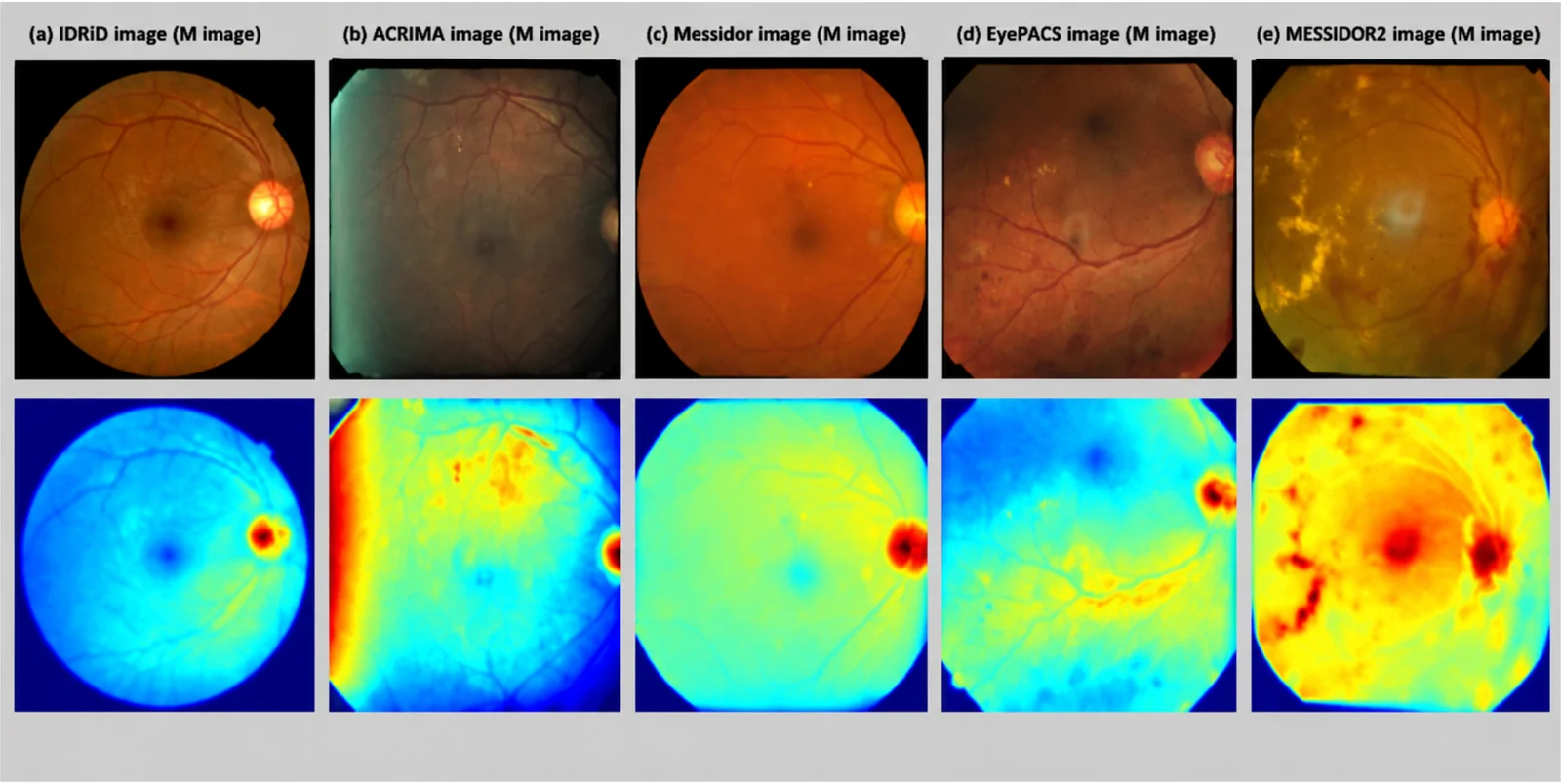
Grad-CAM visualizations across five DR severity levels showing model attention on clinically relevant retinal regions such as microaneurysms, hemorrhages, and neovascularization.

Neither dataset provides lesion-level annotations, so the assessment above is qualitative. Because the heatmaps are computed through the ViT head, they show where the shared backbone finds evidence, not how the tree ensemble weighs that evidence. A quantitative evaluation against pixel-level ground truth is a natural next step.

#### 4.2.3 Comparative analysis and ablation study

A full assessment was performed to compare the ViT-XGB model with CNN, ViT, and ViT-hybrid baselines trained and tested on the same split. As shown in Table 6, the proposed scheme compares favorably with all of these baselines.

To place our results in the wider literature, Table 7 lists accuracies reported by recent studies on APTOS 2019. These values come from the original papers, which used their own splits, preprocessing, and in two cases a binary task, so they provide context rather than a direct comparison. Within our own experiments, ViT-XGB outperformed the second-best configuration, ViT + Random Forest, by 9.05%, and the McNemar bounds in Table 6 show that the differences are unlikely to be due to chance even in the least favorable case. We do not, however, attribute this gap to the choice of XGBoost alone. Differences of this size between classifiers trained on the same features are larger than those usually reported for tree ensembles on tabular data,^16^ and the ablation study below shows that most of the gain comes from the full pipeline: removing SMOTE lowers accuracy to 83.17%, which is below the ViT + Random Forest baseline. Statistical significance on an internal split also does not establish that the advantage will carry over to other populations. Among the published results in Table 7, the two studies with accuracies close to ours^13^^,^^99^ addressed binary detection, which is an easier task than five-class grading, and the remaining studies used APTOS 2019 alone with their own splits. These numbers therefore show that our results are in a plausible range for the task, but they cannot rank the methods.

**Table 6:** Comparative Analysis with Baseline Methods (mean ± standard deviation over five runs). The last column gives an upper bound on the exact McNemar p-value for the comparison with ViT-XGB.

Model	Overall accuracy	Macro F1-Score	Weighted F1-Score	Cohen’s Kappa	McNemar p (upper bound)
DenseNet-121	78.39%±0.72%	75.86%±0.82%	77.68%±0.74%	0.726 ± 0.012	$6.3\times 10^{-33}$
ResNet-50	80.74%±0.67%	78.25%±0.76%	80.09%±0.69%	0.756 ± 0.011	$1.4\times 10^{-28}$
EfficientNet-B4	82.16%±0.62%	79.83%±0.71%	81.47%±0.65%	0.773 ± 0.010	$6.7\times 10^{-26}$
Standalone ViT	85.92%±0.53%	83.74%±0.61%	85.28%±0.56%	0.821 ± 0.009	$4.8\times 10^{-19}$
ViT + SVM	87.83%±0.49%	85.36%±0.58%	87.15%±0.53%	0.845 ± 0.008	$1.0\times 10^{-15}$
ViT + Random Forest	89.47%±0.45%	87.25%±0.52%	88.93%±0.48%	0.864 ± 0.007	$5.8\times 10^{-13}$
**ViT-XGB (Ours)**	98.52%±0.28%	97.72%±0.34%	98.32%±0.25%	0.978±0.005	(n/a)

The ViT features with traditional classifiers (random forest and SVM) led to moderate findings; however, they were better than the findings of the CNN-based methods and below those of our proposed hybrid method. This performance trend is consistent with the complementary nature of transformer-based feature extraction and gradient boosting classification, and with the difficulty that simpler classifiers have with complex patterns and class imbalance in DR grading. The ablation study in Table 8 isolates the contribution of each component of our architecture.

**Table 7:** Accuracies reported by prior studies on APTOS 2019. Values are taken from the original papers and are not directly comparable, because the studies used different splits, preprocessing, and in some cases a different task.

Study	Methodology	Task	Data and split	Accuracy
Alyoubi et al. ^44^	CNN512	5-class	APTOS 2019, own split	84.10%
Youldash et al. ^42^	EfficientNetB3	5-class	APTOS 2019, own split	85.10%
Kobat et al. ^43^	DenseNet201 + Patch Division	5-class	APTOS 2019, 80/20	87.43%
Oulhadj et al. ^46^	ViT + Modified Capsule Network	5-class	APTOS 2019, own split	88.18%
Ahmed et al. ^45^	OcuViT (Vision Transformer)	DR grading	APTOS 2019, own split	95.00%
Folorunsho et al. ^41^	CNN+LSTM+SRNN+XGBoost Ensemble	Multiclass	APTOS 2019, own split	95.63%
Mohanty et al. ^99^	DenseNet121	Binary	APTOS 2019, 80/20	97.30%
Laurensia et al. ^13^	EfficientNet + XGBoost	Binary	APTOS 2019, own split	98.00%
**ViT-XGB (Ours)**	**Vision Transformer + XGBoost + SMOTE**	**5-class**	**APTOS 2019 + Kaggle subset, 80/10/10**	**98.52%**

The ablation findings reveal the important dependencies on which our performance relies and support our architectural design. Without SMOTE, the accuracy decreases by 15.35%, and the minority class recognition is strongly affected (F1-score for Severe DR decreases by 22.45%); this further consolidates the importance of properly managing the label imbalance. Substituting XGBoost with a conventional MLP classifier led to a decrease in performance of 9.77%, which suggests that XGBoost forms more effective decision boundaries for multiclass DR grading on these features. The absence of CLAHE preprocessing led to an accuracy 7.14% lower, which indicates that contrast enhancement helps to bring out the faint DR details. Notably, the direct ViT features obtained only 85.92% accuracy, which is substantially lower than that of our combined method, demonstrating the complementary nature between transformer-based feature extraction and XGBoost classification. The t-SNE dimensionality reduction offered a good compromise in terms of a modest performance change (2.63%) while providing substantial computational advantages; as noted earlier, part of this gain may come from the transductive nature of the embedding. Our findings suggest that the performance of the ViT-XGB framework stems from the combination of complementary components, each handling a particular challenge of the diabetic retinopathy classification problem.

**Table 8:** Ablation Study Results (mean ± standard deviation over five runs). The configuration without t-SNE is fully inductive.

Configuration	Overall Accuracy	Macro F1-Score	Severe DR F1	PDR F1
Full ViT-XGB Model	98.52%±0.28%	97.72%±0.34%	95.90%±0.80%	98.10%±0.50%
Without SMOTE	83.17%±0.63%	80.29%±0.74%	73.45%±1.52%	75.83%±1.38%
Without CLAHE	91.38%±0.41%	89.47%±0.49%	86.25%±1.12%	88.42%±0.98%
Without t-SNE	95.89%±0.32%	94.63%±0.38%	92.18%±0.85%	94.15%±0.73%
Replace XGBoost with MLP	88.75%±0.46%	86.52%±0.54%	83.47%±1.24%	85.83%±1.09%
ViT Features Only	85.92%±0.53%	83.74%±0.61%	80.86%±1.31%	83.07%±1.17%

### 4.3 Discussion

The experimental findings support the proposed ViT-XGB model for multiclass DR severity grading. Under a common protocol, it achieved the highest accuracy among the configurations we tested (98.52%), outperforming the ViT + Random Forest and ViT + SVM baselines by 9.05% and 10.69%, respectively. We read this gap mainly as evidence that the interaction between feature representation, class balancing, and classifier matters when the class distribution is highly skewed, as in DR grading, rather than as a property of the classifier alone.

The learning curves in Fig. 8 flatten after about 35 epochs. At the end of training, accuracy is 0.985 on the training partition and 0.975 on the validation partition, and the loss is 0.120 and 0.180, respectively. The gap of about 1% in accuracy and 0.06 in loss is small, and the validation loss does not rise at any point, so the curves show no sign of overfitting within the 50-epoch budget. Training ran for this fixed budget without early stopping. Because the validation loss reached a plateau and stayed there, an early-stopping rule based on validation loss would have selected a model from the same plateau.

The performance of ViT-XGB can be attributed to several factors. ViT can leverage its self-attention to extract both local lesion features and global contextual relations, enabling it to identify complex co-existing pathologies. This complements the classification power of XGBoost, especially in a high-dimensional feature space, and the built-in regularization that helps mitigate overfitting and improve generalization performance of the model. Most importantly, feature-space SMOTE gives the classifier a balanced training set, and the 15.35% drop in accuracy without it shows how much the label imbalance would otherwise cost. The model’s discriminative ability is further illustrated in Fig. 9, which shows the ROC curves for all severity levels of the disease. The class-wise AUCs and their 95% CIs are listed in Table 5. The consistently high performance in all classes supports a balanced diagnostic accuracy for the entire range of severity.

**Fig. 8:**
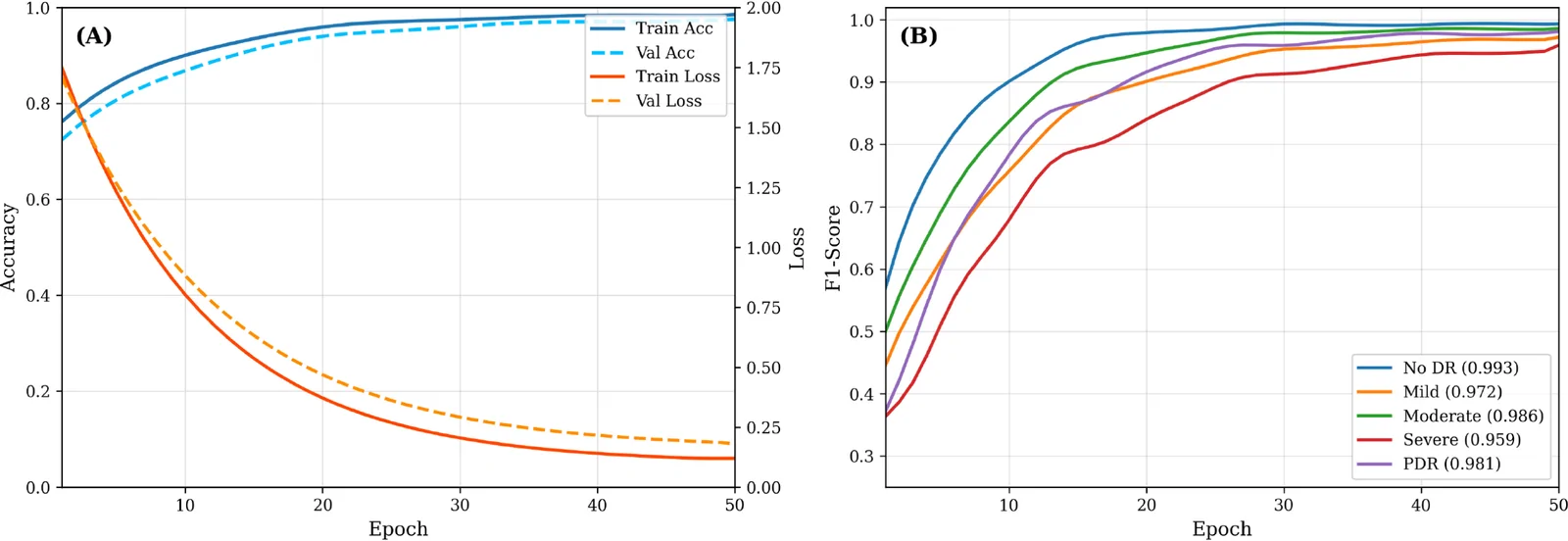
Training and validation curves of the ViT-XGB model showing (A) overall accuracy and loss progression, and (B) class-wise performance across DR severity classes. Final values are given in the legends; the training and validation curves are plotted on the same axes to show the gap between them.

The Grad-CAM maps indicate that the model attends to clinically recognized features: microaneurysms for Mild DR, hemorrhages of various types for Moderate DR, venous abnormalities for Severe DR, and neovascularization for PDR. As discussed above, this assessment is qualitative, and a pixel-level evaluation against annotated lesion masks is still needed. Interpretability is crucial for establishing clinical trust and promoting adoption in the healthcare sector. The ablation study provides guidance for real-world use, including the necessity of SMOTE for the label imbalance problem and the stronger classification ability of XGBoost compared with that of other algorithms on these features. The small effect of t-SNE (2.63% performance change) implies that even though dimensionality reduction brings computational benefits, the proposed model remains accurate with high-dimensional features, as long as sufficient computational resources are provided.

**Fig. 9:**
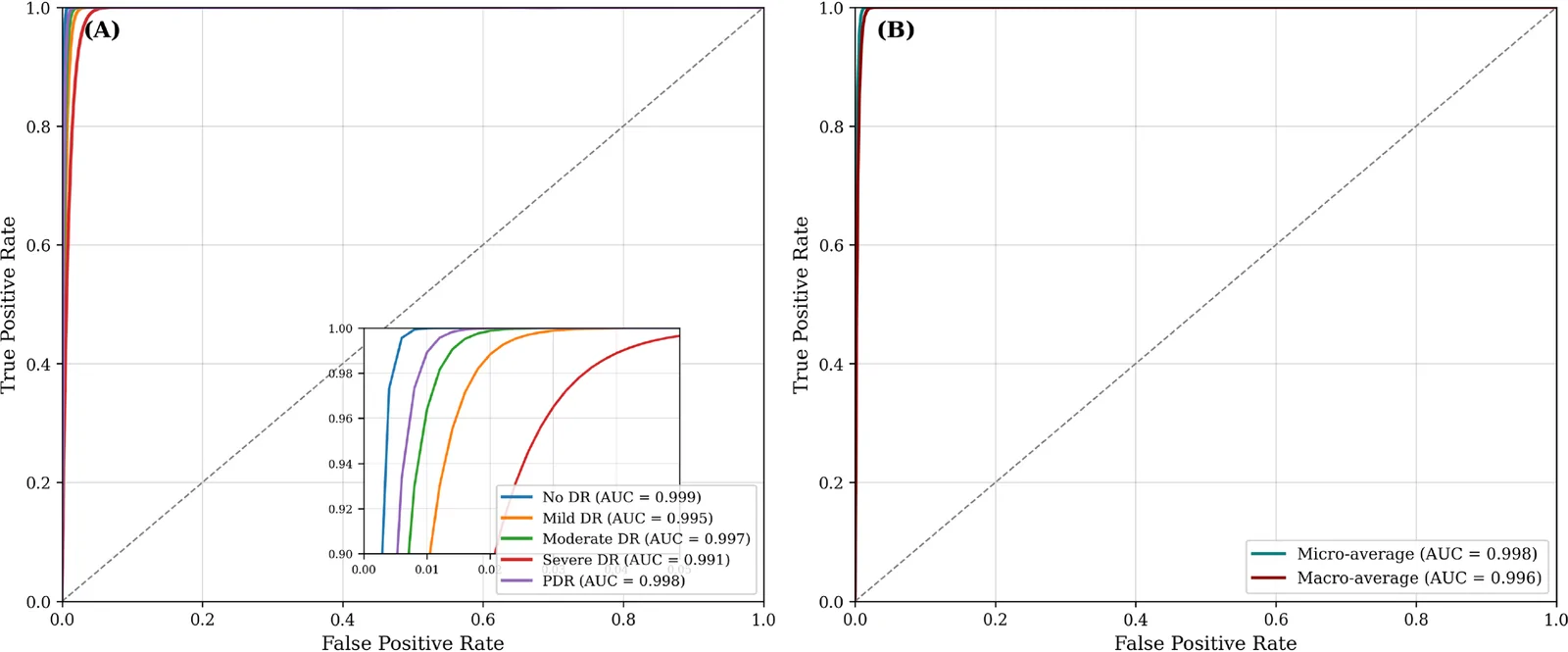
Multiclass ROC analysis of the ViT-XGB model showing (A) class-wise ROC curves with individual AUC values, and (B) overall micro- and macro-average ROC curves reflecting the aggregated classification performance. The 95% CIs of the class-wise AUCs are given in Table 5.

There are some factors that may affect the generalizability of these results, and we list them so that the findings can be read in context. First, the test images come from the same two sources as the training images. The split therefore measures performance under matched acquisition conditions rather than under the shift that a new clinic, camera, or population would introduce.^100^ Second, the split was made at the image level. EyePACS provides images of both eyes of each patient and APTOS 2019 does not release patient identifiers, so images of the two eyes of one person may fall into different partitions. Fellow eyes tend to share image characteristics and disease stage, which can raise test accuracy. Third, the t-SNE stage is transductive; the fully inductive variant reached 95.89%, which we regard as a more conservative estimate for single-image deployment. Fourth, the test set contains only 49 Severe DR and 30 PDR images, so the estimates for these grades carry wide intervals; for PDR, the 95% CI for sensitivity spans 85.3% to 99.8%. Finally, accuracy well above the level of agreement between individual graders^81^ should be read as agreement with the dataset labels, not as evidence that the model exceeds clinical experts. Independent validation on external data is the only way to settle these points.

### 4.4 Computational Cost and Deployment

Table 9 summarizes the cost of each stage. The ViT forward pass dominates inference, at about 17.6 billion multiply-accumulate operations per image, while the tree ensemble adds at most 20,000 threshold comparisons (2500 trees of depth at most eight), which is negligible. Training is more demanding. Fine-tuning ViT-B/16 for 50 epochs on 5523 images involves roughly $1.5\times 10^{16}$ multiply-accumulate operations for the forward and backward passes and needs a GPU. Exact t-SNE on about 17,000 vectors stores an affinity matrix of about 2.3 GB in double precision and costs $O\left(n^{2}d\right)$ operations per iteration. More importantly for deployment, t-SNE has no transform for new images, so a deployed system should use the inductive variant or a parametric embedding. Because the ViT forward pass accounts for nearly all of the inference cost, CPU-only deployment is feasible in principle for screening workloads that do not need real-time throughput, and 8-bit quantization would reduce the model to about 86 MB. Wall-clock latency depends strongly on the hardware and software stack, and we did not benchmark it on CPU-only or edge devices; this is part of the planned deployment study.

**Table 9:** Computational profile of the ViT-XGB pipeline (hardware-independent quantities).

Stage	Size	Cost per image	Remarks
Preprocessing	No learned parameters	A few passes over the pixels	Masking, CLAHE, resizing
ViT-B/16	85.8 M parameters; 343 MB (fp32)	About 17.6 GMACs	Dominant inference cost
t-SNE (d=45)	Non-parametric	Not defined for a single new image	Transductive; replace with a parametric map or omit
XGBoost	2500 trees, depth ≤8	At most 20,000 comparisons	Negligible
Grad-CAM (optional)	Shares ViT weights	One forward and one backward pass	Generated on demand

### 4.5 Clinical Translation, Limitations, and Future Directions

Our results support ViT-XGB as a candidate decision-support tool, not as a system ready for autonomous clinical use. Three steps separate the two. The first is validation on independent data. External datasets such as Messidor-2,^101^ IDRiD,^102^ and DDR,^103^ as well as the EyePACS images not used here, would test robustness to new cameras and populations without retraining. A prospective study reported according to TRIPOD+AI^104^ or CONSORT-AI^105^ would then be needed to measure real-world performance. The second is comparison with clinicians. We did not run a reader study, so we cannot say how the model compares with ophthalmologists or trained graders on the same images. A study that grades the same test images by several readers against an adjudicated reference standard^81^ is part of our planned work. The third is regulatory clearance. Software that grades DR is regulated as a medical device. The autonomous IDx-DR system received De Novo authorization from the US Food and Drug Administration after a prospective pivotal trial,^106^ and EyeArt was cleared after a similar multicenter evaluation.^107^ In Europe, such software requires CE marking under the Medical Device Regulation.^108^

Until such evidence exists, we envisage the model operating under human oversight. Every referable or low-confidence output would be reviewed by a qualified grader, who remains responsible for the clinical decision, as with current assistive tools. Several failure modes can be anticipated. Images with blur, uneven illumination, or small pupils reduce the visibility of lesions, and the model has no quality gate, so it will grade such images anyway. Lesions that shrink to one or two pixels after resizing, mainly isolated microaneurysms, are the most likely to be missed, which fits the confusions we observe between No DR and Mild DR. Adjacent grades, especially Moderate and Severe NPDR, are the most frequent source of error (Fig. 6). Other retinal diseases, laser scars from earlier treatment, and images from cameras or populations absent from training fall outside what the model has learned. Because the datasets carry no image-quality or comorbidity labels, we could not quantify performance by image type. In practice, a quality check before grading and an uncertainty estimate after it, with referral of uncertain cases, would contain most of these risks.

Demographic representation is another open question. APTOS 2019 was collected in India, and the Kaggle images come from the EyePACS program, which operates mainly in primary care clinics in the United States.^73^ Neither dataset releases age, sex, or ethnicity, so we could not measure performance by demographic subgroup. This matters because fundus pigmentation and retinal appearance vary across populations, and models trained on one population can lose accuracy on another.^109^^,^^110^ Subgroup analysis on datasets that include demographic metadata is needed before the model is used outside the populations represented here. Both datasets were released in de-identified form for research, and this study used no other patient data. Clinical deployment would change the situation. Fundus photographs are personal health data under regulations such as HIPAA in the United States and the GDPR in Europe, so a deployed system would need secure handling of images, clear information for patients, and consent procedures that meet local requirements.

The reported results come from one fixed split repeated with five seeds, which captures variability from training but not from the choice of split. Building on this work, we plan to (i) validate the model externally on the datasets listed above; (ii) repeat the evaluation with patient-level, stratified k-fold cross-validation where patient identifiers are available; (iii) compare SMOTE with ADASYN, Borderline-SMOTE, and class weighting, t-SNE with PCA and UMAP, and XGBoost with LightGBM and CatBoost, on the same features; (iv) replace t-SNE with a parametric embedding so that single images can be graded inductively; (v) test higher input resolutions and retina-specific pretraining such as RETFound; (vi) benchmark latency on GPU, CPU, and edge hardware; (vii) add uncertainty quantification so that uncertain cases are routed to human graders; and (viii) conduct a reader study with ophthalmologists. The modular design of the framework makes these extensions straightforward while preserving the core of the ViT-XGB method.

## 5 Conclusion

We proposed ViT-XGB, a framework that combines Vision Transformer feature extraction with an XGBoost classifier for five-class diabetic retinopathy grading. On an internal test set of 691 images drawn from APTOS 2019 and a subset of the Kaggle 2015 EyePACS data, it reached an accuracy of 98.52% (95% CI, 97.31% to 99.19%), with balanced performance across the five severity levels, and it compared favorably with CNN, ViT, and ViT-hybrid baselines trained under the same protocol. The main ingredients are the pairing of ViT features with gradient boosting, class balancing with SMOTE in the feature space of the training data, and Grad-CAM heatmaps that expose the retinal regions behind each prediction. Inference cost is dominated by a single ViT forward pass, while the t-SNE stage should be replaced by an inductive mapping for deployment. The results are encouraging for screening and teleophthalmology, but they come from data that share their sources with the training set. External validation on independent, demographically diverse cohorts, a comparison with human graders, and prospective evaluation are the next steps toward clinical use.

## CRediT authorship contribution statement

**Azhar Imran:** Writing – original draft, Methodology, Conceptualization. **Sun-Hee Kim:** Writing – review & editing, Validation, Investigation, Formal analysis. **Jahanzaib Latif:** Visualization, Software, Data curation. **Raja Majid Mehmood:** Writing – review & editing, Supervision, Resources, Investigation.

## Ethics declaration

The authors have NOT obtained informed consent from participants or their legal representatives. Verbal consent was not obtained because this study involved secondary analysis of publicly available, de-identified retinal fundus image datasets (APTOS 2019 Blindness Detection and Kaggle Diabetic Retinopathy Detection datasets). No new data were collected, no participants were recruited, and no direct interaction with individuals occurred. The datasets contain no personally identifiable information; therefore, obtaining individual informed consent was not applicable for this study.

This study was performed in compliance with relevant laws, regulatory frameworks and guidelines where the research took place. Ethics committee approval was not required under relevant laws and institutional guidelines. This study used publicly available, de-identified retinal fundus images from the APTOS 2019 Blindness Detection and Kaggle Diabetic Retinopathy Detection datasets. No new human data were collected, and no identifiable patient information was accessed. Therefore, ethics committee approval was not required for this secondary analysis in accordance with institutional guidelines and regulations governing the use of publicly available anonymized datasets.

This study used only publicly available, de-identified fundus images from the APTOS 2019 Blindness Detection and Kaggle 2015 Diabetic Retinopathy Detection datasets, which were released for research under the terms of the respective competitions. No new images were collected and no patients were contacted, so ethical approval and individual consent were not required for this secondary analysis.

## Declaration of competing interest

The authors declare that they have no known competing financial interests or personal relationships that could have appeared to influence the work reported in this paper.

## Data Availability

The APTOS 2019 dataset is available at https://www.kaggle.com/competitions/aptos2019-blindness-detection, and the Kaggle 2015 dataset is available at https://www.kaggle.com/competitions/diabetic-retinopathy-detection. The list of Kaggle image identifiers used in this study and the split assignment of every image will be released with the code.
